# Pigment Dispersing Factor Is a Circadian Clock Output and Regulates Photoperiodic Response in the Linden Bug, *Pyrrhocoris apterus*

**DOI:** 10.3389/fphys.2022.884909

**Published:** 2022-04-29

**Authors:** Joanna Kotwica-Rolinska, Milena Damulewicz, Lenka Chodakova, Lucie Kristofova, David Dolezel

**Affiliations:** ^1^ Institute of Entomology, Biology Centre of the Czech Academy of Sciences, České Budějovice, Czech Republic; ^2^ Institute of Zoology and Biomedical Research, Jagiellonian University, Kraków, Poland; ^3^ Faculty of Science, University of South Bohemia, České Budějovice, Czech Republic

**Keywords:** pigment dispersing factor, cryptochrome-m, circadian clock, photoperiodic clock, diapause, CRISPR/Cas9, Pyrrhocoris apterus

## Abstract

Daily and annually cycling conditions manifested on the Earth have forced organisms to develop time-measuring devices. Circadian clocks are responsible for adjusting physiology to the daily cycles in the environment, while the anticipation of seasonal changes is governed by the photoperiodic clock. Circadian clocks are cell-autonomous and depend on the transcriptional/translational feedback loops of the conserved clock genes. The synchronization among clock centers in the brain is achieved by the modulatory function of the clock-dependent neuropeptides. In insects, the most prominent clock neuropeptide is Pigment Dispersing Factor (PDF). Photoperiodic clock measures and computes the day and/or night length and adjusts physiology accordingly to the upcoming season. The exact mechanism of the photoperiodic clock and its direct signaling molecules are unknown but, in many insects, circadian clock genes are involved in the seasonal responses. While in *Drosophila*, PDF signaling participates both in the circadian clock output and in diapause regulation, the weak photoperiodic response curve of *D. melanogaster* is a major limitation in revealing the full role of PDF in the photoperiodic clock. Here we provide the first description of PDF in the linden bug, *Pyrrhocoris apterus*, an organism with a robust photoperiodic response. We characterize in detail the circadian and photoperiodic phenotype of several CRISPR/Cas9-generated *pdf* mutants, including three null mutants and two mutants with modified PDF. Our results show that PDF acts downstream of CRY and plays a key role as a circadian clock output. Surprisingly, in contrast to the diurnal activity of wild-type bugs, *pdf* null mutants show predominantly nocturnal activity, which is caused by the clock-independent direct response to the light/dark switch. Moreover, we show that together with CRY, PDF is involved in the photoperiod-dependent diapause induction, however, its lack does not disrupt the photoperiodic response completely, suggesting the presence of additional clock-regulated factors. Taken together our data provide new insight into the role of PDF in the insect’s circadian and photoperiodic systems.

## Introduction

The Earth’s rotation around its axis and Earth’s orbit around the Sun enforce regular predictable daily and annual changes in the environment. As a consequence, organisms had to develop ways to cope with the cycling of abiotic and biotic factors and evolved circadian and photoperiodic clocks as a response to the 24 h and seasonal periodicities, respectively. Circadian clocks are endogenous mechanisms allowing for accurate adjustment to the daily changes in the light-dark cycles. The clock perceives environmental stimuli by the set of photo- and thermo-receptors and *via* input pathway transmits the information to the oscillator. The mechanism of the oscillator is based on the transcriptional-translational feedback loops and in animals the function of most of the genes encoding the molecular machinery of the circadian clock is conserved. In *Drosophila* two transcription factors Clock (CLK) and cycle (CYC) form a heterodimer CLK/CYC and activate expression of the *period* (*per*) and *timeless* (*tim*) genes. PER/TIM heterodimer in turn blocks the activity of CLK/CYC and inhibits transcription of its own genes – forming the negative feedback loop. During the day, activation of the light-sensitive cryptochrome (CRY-d) leads to TIM degradation, followed by degradation of PER and disinhibition of the CLK/CYC. This allows the cycle to start over (reviewed in (Hardin, 2011; Tomioka and Matsumoto, 2019; Beer and Helfrich-Forster, 2020). In other insect species, the role of clock genes is mostly conserved, however the detailed architecture of the circadian clock differs from *Drosophila*. Recently we have shown that while the CLK/CYC heterodimer retains its role in a positive feedback loop in *Pyrrhocoris apterus*, the negative feedback loop consists of non-photosensitive CRY-m and possibly PER, but TIM is not essential for the function of the clock (Kotwica-Rolinska et al., 2021). Additionally, *P. apterus*, similarly to several other insects, lacks photosensitive CRY-d (reviewed in (Tomioka and Matsumoto, 2015; Beer and Helfrich-Forster, 2020). Therefore, in contrast to *Drosophila*, the light input pathways to the clock are possibly exclusively dependent on the rhodopsin-based photoreceptors in *P. apterus*.

The information about the external time is interpreted by the circadian clock in the brain and transferred by the output pathways synchronizing the activity of the whole organism. In insects, several neuropeptides play the role in the circadian clock output and Pigment Dispersing Factor (PDF) is a key factor in this process (reviewed in (Shafer and Yao, 2014; Hermann-Luibl and Helfrich-Forster, 2015). In the majority of insects, including *Drosophila*, cells producing PDF in the brain are located exclusively in the anterior medulla region and based on anatomical localization are described as lateral ventral neurons (LNv) (Sehadova et al., 2003; Ikeno et al., 2014; Koide et al., 2021) and reviewed in (Shafer and Yao, 2014; Hermann-Luibl and Helfrich-Forster, 2015), however, in several hemimetabolan insects, additional cells located in the lamina are found (Sato et al., 2002; Lee et al., 2009; Gestrich et al., 2018). In *Drosophila*, distinct groups of PDF-expressing LNvs send neuronal projections to the medulla (large – l-LNv), the dorsal protocerebrum (Dp) (small – s-LNv), and both, s-LNv and l-LNv innervate the accessory medulla (aMe), a small neuropil regarded as insects master pacemaker (Helfrich-Forster, 1995, 1998; Helfrich-Forster et al., 2007). s-LNvs are the main group responsible for the synchronization of other clocks located in the brain and have shown rhythmic production of PDF in the cells somata and its rhythmic release in the dorsal protocerebrum arborizations (Park et al., 2000; Shafer and Taghert, 2009). *Drosophila* mutants lacking PDF (Renn et al., 1999) or PDF receptor (Hyun et al., 2005; Lear et al., 2005; Mertens et al., 2005) become gradually arrhythmic in constant darkness (DD) proving that PDF is the main neuropeptide involved in the circadian output system. While currently, *Drosophila* is the only insect in which the role of PDF was studied with the help of genetic manipulations, in other insects PDF was proven to participate in the circadian clock output pathways by means of active peptide injections (Petri and Stengl, 1997; Singaravel et al., 2003; Beer et al., 2018) or downregulation of expression by RNAi (Lee et al., 2009; Hassaneen et al., 2011). Therefore, it seems that the role of PDF in insects’ circadian clocks is conserved.

The photoperiodic clock is a device that allows insects to anticipate and adapt to seasonal changes during the year. The main cue is the changing photoperiod during a year and the photoperiodic clock adjusts the organisms’ physiology based on the measurement of the length of a day or night (reviewed in (Saunders, 2020)). The circadian clock, as a proven time measuring device, is also predicted to play a role in time measurement in the photoperiodic clock. While the actual mechanism linking the circadian clock and photoperiodism is still under debate it was already shown that circadian clock genes are involved in the photoperiodic response in many insect species. Downregulation by RNAi of *per* (Ikeno et al., 2010) and *cry-m* (Ikeno et al., 2011) induces reproduction under short-day conditions (SD) in *Riptortus pedestris*. On the other hand downregulation of the positive elements of the circadian clock – CLK and CYC inhibits reproduction under long photoperiod (LD) in *R. pedestris* and *P. apterus* (Ikeno et al., 2010; Kotwica-Rolinska et al., 2017). Interestingly, the opposite situation is found in the monarch butterfly, *Danaus plexippus*, where knock-out of the *cry-m* induces diapause in LD conditions and knock-out of either *Clk* or *cyc* increases egg laying in SD conditions (Iiams et al., 2019). Although the genetic manipulation of circadian clock genes results in either diapause or reproduction in a species-specific manner, both outcomes support the involvement of the circadian clock machinery in the photoperiodic response of insects. How could the circadian clock regulate the function of the photoperiodic clock? One possibility is that neuropeptides, such as PDF, which are involved in the output pathway of the circadian clock, also play a role as signaling molecules transferring information about the day length downstream to the target cells and tissues. Currently, the role of PDF in the response to the photoperiod was shown for a few species but the specific role of the PDF differs among insects. In *Drosophila*, *pdf* ^*01*^ mutants show increased reproductive dormancy in long and short photoperiod, while *pdf* overexpression decreases reproductive dormancy (Nagy et al., 2019). Similarly in the mosquito *Culex pipens*, depletion of *pdf* induced the diapause in long photoperiod (Meuti et al., 2015). In contrast, downregulation of *pdf* by RNAi in *Plautia stali* reduced the level of the imaginal diapause in SD (Hasebe et al., 2022) but the same procedure did not change the reproductive status of females either in short or in long-day conditions in *R. pedestris* (Ikeno et al., 2014). The involvement of PDF in the seasonality was indicated in a whole-genome study of the European corn borer moth (*Ostrinia nubilalis*), where PDF receptor was identified in the genomic region responsible for the early and late emergence in spring (Kozak et al., 2019).

The linden bug *P. apterus* is a long-established model for studies concerning diapause and photoperiodism (reviewed in (Socha, 1993; Saunders, 2021) and references therein), and recently also the circadian clock in this species was addressed in more detail (Pivarciova et al., 2016; Kaniewska et al., 2020; Kotwica-Rolinska et al., 2021). Linden bugs are typical diurnal animals showing the peak of the activity around the middle of the day. When transferred to the constant conditions they stay predominantly rhythmic, however the percentage of the rhythmic individuals and the length of the free-running period (τ) is dependent on the geographical origin of the line (Pivarciova et al., 2016). *P. apterus* overwinters in the state of the “reproductive diapause,” which is characterized by the cessation of the ovarian development and egg production, decrease in overall activity, an increase of the storage proteins and lipids, and cold hardening (Socha, 1993; Kostal and Simek, 2000). *P. apterus* shows a clear photoperiodic response, being reproductive in the long-day conditions and arrests reproduction under short-day conditions even at a relatively high temperature of 25°C (Saunders, 1983).

The recent development of the genome-editing tools like CRISPR/Cas9 which allows for relatively easy creation of mutants pushes forward the knowledge of the mechanism of the circadian and photoperiodic clock in insects other than *Drosophila*. Here we describe the phenotype of the *pdf* null mutants in the linden bug, *P. apterus*, the first available mutant in this gene in the non-model insect. The behavior of *P. apterus pdf* null mutants is remarkably different than *Drosophila*, showing predominantly nocturnal activity in LD cycles and instant arrhythmicity in constant conditions. While the arrhythmicity can be explained by the effect of *pdf* mutation on dysregulation of expression of the circadian clock genes, the genetic manipulation of clock genes does not mirror *pdf* null nocturnal activity. Although currently the mechanism of this nocturnal activity has not been identified, our results suggest that it is a clock-independent direct response to the light:dark switch. Moreover, we show that CRY-m and its output PDF play a role as a diapause-inducing factor. In contrast to CRY-m, PDF is not completely necessary for the photoperiodic response, suggesting an involvement of additional possibly clock-regulated factors. Taken together, our data provide new insight into the PDF role in the insects’ circadian and photoperiodic systems.

## Materials and Methods

### Insects

The colony of *P. apterus* was kept in long-day conditions (LD) (photoperiod 18 h light and 6 h darkness and constant temperature 25°C) with access to linden seeds (*Tilia cordata*) and water *ad libitum*. Roana strain described in Pivarciova et al. (2016) was used for genome editing, mutant lines backcrossing, and served as a wild-type (WT) reference. Part of experiments was performed on *cry* null mutants: *cry-m* ^*04*^ and *cry-m* ^*9in*^ mutants described in Kotwica-Rolinska et al. (2021). All experiments performed on male bugs were performed on animals from colonies kept in LD. Experiments on females were performed on animals grown in LD or short-day conditions (SD) (photoperiod 12 h light and 12 h darkness and constant temperature 25°C).

### CRISPR/Cas9 Genome Editing

*pdf* sequence was obtained from in-house transcriptomic and genomic databases. Predicted PDF prohormone signal peptide, cleavage sites, and PDF posttranslational modification were established with the use of SignalP 5.0 and Neuropred online tools (Southey et al., 2006; Almagro Armenteros et al., 2019). The detailed methodology of *P. apterus* gene editing is described in Kotwica-Rolinska et al. (2019). In short, two distinct guide RNAs targeting genomic sequence encoding PDF active peptide were used (*see* Supplementary Figure S1 for the *pdf* sequence and position of guides). Embryos were injected with gRNAs mixed with the commercial Cas9 protein (CP01 from PNA Bio) as described previously (Kotwica-Rolinska et al., 2019). G0 adults were mated to the wild-type bugs. In the next generation, individuals with successfully modified *pdf* gene were identified from antennal-squish PCR. Ten to 15 generations of backcrosses to WT strain were used to outcross possible off-target modifications.

### Locomotor Activity

In the initial experiments, the following protocol was applied: heterozygotes were crossed together, and their adult male progeny (which consisted of the mixture of wild-type, heterozygotes, and homozygotes) were used to perform locomotor activity run. After the run ended and analyses of the behavior were performed, individuals were genotyped by PCR. This setup allowed us to show the true effect of *pdf* mutation and discern the obtained phenotype from possible off-target and the bottleneck effect. In this study, we use WT abbreviation to point experiment performed on parental Roana strain and *pdf* ^*+/+*^, *pdf* ^*+/−*^ and *pdf* ^*−/−*^ to describe the phenotype of wild-type, heterozygotes, and homozygous mutants derived from crosses.

Activity analysis was performed on males 3–5 days after adult ecdysis. Bugs were individually placed in the LAM (Large Activity Monitors, Trikinetics, Inc., Waltham, MA, United States), supplemented with food and water *ad libitum*. The activity was recorded with 5 min bins during the entire experiment. Bugs were entrained for 5 days in light-dark conditions, followed by 12 days in constant conditions (constant temperature with either constant darkness or constant light). In experiments involving locomotor activity in different photoperiods and experiments with the use of RNAi the time of entrainment in the light-dark conditions was set up for 10 days. Light intensity in LD and LL experiments was set up to ∼400 lx (3 W/m^2^). All activity measurements were performed in the Cooled Incubator Sanyo MIR-154 equipped with a built-in electronic timer. The actual light intensity and temperature were recorded by *Drosophila* Environmental Monitors (Trikinetics, Inc., Waltham, MA, United States).

All the activity was analyzed in the ActogramJ plugin of the FiJi software (Schmid et al., 2011). Activity analysis in LD cycles was performed as follows. Activity of every bug was analyzed with the “average activity” tool with the “Period” set up for 7200 min (5 days) or 14,400 min (10 days) with the “Smoothing Gaussian std dev” value set to 20 min. If the bug showed one activity peak in the 24 h period in all consecutive days, it was scored as “rhythmic in LD.” If several irregular peaks of activity in the 24 h period were detected and no clear diel rhythm of activity was observed - bugs were scored as “arrhythmic in LD.” Examples showing rhythmic and arrhythmic bugs are provided in Supplementary Figure S1. Daily activity graphs represent the mean activity of all bugs scored as rhythmic in LD and are calculated based on raw, unsmoothed measurements. In the case of the activity graphs presented in Figure 4, we used activity calculated with “Smoothing Gaussian std dev” with the value set to 20 min. For the analysis of the shift in the activity, we first calculated smoothed values as described above. Then, we manually calculated the onset and offset, which were defined as a time when bugs reached the median value of the average total activity during the day. To assess rhythmicity and free-running period (τ) of bugs in constant conditions, the Lomb Scargle periodogram was used. Based on the activity pattern in constant conditions, animals were scored as rhythmic, complex, and arrhythmic and τ was calculated only for rhythmic animals, as described previously (Pivarciova et al., 2016; Kaniewska et al., 2020; Kotwica-Rolinska et al., 2021).

### Gene Expression Analysis

The gene expression analysis was performed on bugs kept in LD and SD conditions. In both conditions, ZT0 applies to the light on. Light off in LD occurs at ZT18 and in SD at ZT12. mRNA quantification was performed on RNA isolated from five heads in each sample collected always on the 10th day after adult ecdysis. To check daily changes of gene expression in the photoperiod LD 18:6, heads were collected from WT and *pdf* ^*04 −/−*^ homozygous mutant males every 4 h throughout the day (for the detailed nomenclature of mutants, *see* the Results section). To compare the expression pattern of genes in other mutant strains, heads were collected from WT and *pdf* ^*RK−/−*^, *pdf* ^*05−/−*^, *pdf* ^*04−/−*^ and *pdf* ^*07−/−*^ mutants males at ZT8 and ZT20 (8 h after light on and 4 h before the light on). To analyze the expression of *pdf* in *cry-m* mutants, heads were collected from males kept in LD at ZT8. To compare *pdf* expression between diapause and reproduction inducing conditions, heads of females kept in the SD 12:12 or LD 18:6 were collected at ZT8 and ZT20 (8 h after light on and 4 h before the light on for both conditions). Total RNA was extracted with Trizol reagent (Thermo Fisher Scientific), treated by DNase I (Thermo Fisher Scientific), purified by sodium acetate/ethanol precipitation, and measured on the NanoDrop (Thermo Fisher Scientific). 1 μg of the total RNA was reverse transcribed with Superscript III (Thermo Fisher Scientific) using oligo dT primers, following manufacturer instruction. RT-qPCR was run on qPCR 2× SYBR Master Mix (Top-Bio, Czech Republic) on Bio-Rad CFX96 qPCR instrument (Bio-Rad). Primer specificity was tested beforehand and PCR products were confirmed by sequencing. Primer efficiency was evaluated by an RT-qPCR method on a five-point standard curve. *rp49* was used as a reference gene in all experiments. A list of primer sequences is available in Supplementary Table S1.

### Immunohistochemistry

Brains for the immunohistochemistry were collected always on the 10th day after adult ecdysis. To asses daily changes of PDF staining, brains were dissected from WT males kept in LD 18:6, every 4 h throughout the day. To analyze PDF level in *cry-m* mutants, brains were dissected from males kept in LD at ZT8 and ZT20. To compare PDF levels between SD and LD conditions, the brains of WT females from both conditions were collected at ZT8 and ZT20. Brains were dissected in phosphate-buffered saline (PBS) and then fixed in 4% ice-cold paraformaldehyde (PFA) for 8 h. After five subsequent washing in PBS supplemented with 0.3% TX100 (PBST), brains were blocked with 5%NGS (Normal Goat Serum – Thermo Fisher) in PBST. Incubation with the mouse monoclonal anti- *Drosophila* PDF primary antibody (PDF C7 Developmental Studies Hybridoma Bank) diluted 1:1000 in 5% NGS in PBST was carried out for 2 days at 4°C. After subsequent washes in PBST samples were incubated with the solution of 1:1000 goat-anti-mouse Alexa Fluor 488 fluorescent secondary antibody (Thermo Fisher Scientific) for 2 days at 4°C. After final washings in PBST brains were mounted in the Vectashield mounting medium (Vector Laboratories). The samples were imaged under Laser Scanning Confocal Microscope FluoView FV1000 (Olympus) using objectives UPLSAPO ×10 or UPLSAPO 20xO. For analysis of the changes of PDF staining intensity, confocal settings (photomultiplier sensitivity, laser strength, aperture widths) were kept constant for all images captured. All confocal images were processed and analyzed by ImageJ software (NIH). For densitometry analysis confocal images were merged and transformed to grayscale. The fluorescence intensities of single cells were measured as mean gray values of the whole cell bodies area and background values nearby the cells were extracted from measurements. Staining intensities of PDF projections in dorsal protocerebrum were quantified as mean gray values of the whole area containing PDF projections. The same area in one hemisphere (rectangle 275 μm × 180 μm) containing exclusively PDF Dp projections was selected. The pixel intensity of the entire specified area was determined. A value of pixel intensity of rectangle (20 μm × 20 μm) located outside of PDF projections was used to subtract background signal. The staining intensity for each brain was calculated as an average intensity of staining calculated from both hemispheres.

### RNAi Experiments

Fragments located within the open reading frame of each, *tyrosine hydroxylase* (*TH*) and *Dopamine transporter* (*DAT*) were amplified using PCR, cloned into pGEM-T Easy (Promega), and inserts were verified by Sanger sequencing (the sequences of primers used are listed in Supplementary Table S1). Templates for dsRNA *in-vitro* synthesis were prepared from pGEM-T Easy clones as described previously (Kotwica-Rolinska et al., 2017; Kotwica-Rolinska et al., 2021). As a negative control, *beta-galactosidase* (*lacZ*) dsRNA was used. Adult males were injected with 2 µl of dsRNA at a concentration of 4 mg/ml in Ringer’s solution. Males were injected with dsRNA on the second day after adult ecdysis and together with controls were immediately placed in the Locomotor Activity Monitors. For the assessment of the efficiency of the gene expression downregulation by RNAi, males were injected with dsRNA on the second day after adult ecdysis, and heads of control and experimental animals were collected at ZT8 on the fourth day after injection. The efficiency of the dsRNA treatment was evaluated by RT-qPCR, as described in the Materials and Methods section “Gene expression analysis.”

### Diapause Phenotype

Heterozygotes of each of *pdf* mutant lines and *cry-m* lines were crossed together, and later, their adult female progeny (*pdf* ^*+/+*^, *pdf* ^*+/−*^ and *pdf* ^*−/−*^, and *cry-m* ^*+/+*^, *cry-m* ^*+/−*^ and *cry-m* ^*−/−*^) was used to assess the diapause phenotype. Experimental bugs were transferred to the SD conditions at the early developmental stages (nymphal stage 3). After adult ecdysis, bugs were kept separately in Petri dishes supplied with linden seeds and water, and the appearance of eggs was checked every second day. After 2 weeks, bugs that did not lay eggs were sacrificed and the development of ovaries was additionally determined. Females which did not lay eggs or lacked mature eggs or vitellogenic oocytes were considered to be diapausing. After assessment of reproductive status, all individuals were genotyped by antenna-squish PCR.

### Photoperiodic Response Curve

*P. apterus* from the WT line and homozygotes of *pdf* and *cry-m* mutant lines were transferred in the early developmental stages (nymphal stage 3) to different constant photoperiodic conditions, ranging from 18 h light: 6h darkness to 8 h light: 16 h darkness at 25°C. Diapause phenotype of females was evaluated as described above. At least 30 female bugs were checked for each genotype and each photoperiodic condition and all measurements were repeated at least twice. Altogether, the diapause phenotype was analyzed for over 4,100 animals. For calculation of the critical day length (CDL) the non-linear regression method was used.

### Statistical Analysis

Most of the statistical analyses were performed in Graphpad software. Grubbs’ test was used for outliers’ detection. The D’Agostino-Pearson normality test was used to assess the distribution of the data, followed by an appropriate statistical test. Daily rhythmicity of gene expression and PDF staining was analyzed by the Kruskal-Wallis test and JTK_Cycle software in R (Hughes et al., 2010). Data were analyzed using one-way or two-way ANOVA with *post-hoc* Tukey’s test or *t*-test to compare two groups. The onset and offset analysis was performed in R (CircStats package version 0.2–6) (Agostinelli and Lund, 2018). The time was converted to radians, Rayleigh test was used to analyze the circular distribution of data, and Watson’s U^2^ test was used for statistical comparison between experimental groups. Details about statistics used in different experiments are presented in the Results section.

## Results

### PDF Localization

**FIGURE 1:**
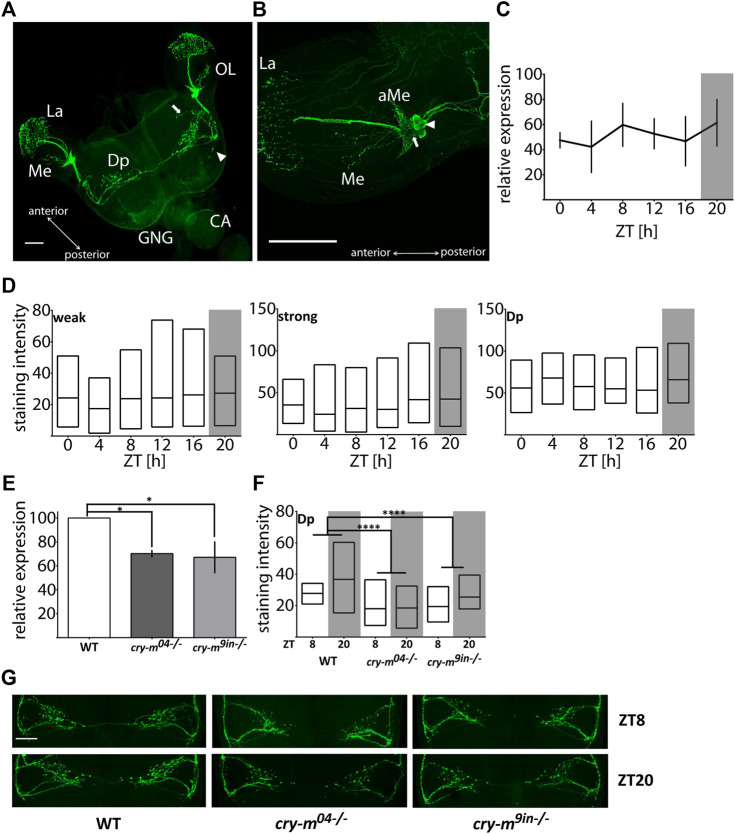
Analysis of the PDF mRNA and protein levels in WT and *cry-m* LD males. **(A)** Localization of PDF in *P. apterus* brain (*see* the text for detailed description). Arrow points to the neuronal branch extending from the anterior dorsal fiber and arrowhead points to the neuronal extension of the posterior dorsal fiber. **(B)** Localization of PDF in the optic lobe. Two groups of cells can be distinguished: weakly (arrow) and strongly (arrowhead) stained cells. OL, optic lobe; GNG, gnathal ganglion; CA, corpora allata; La, Lamina; Me, medulla; Dp, dorsal protocerebrum; aMe, accessory medulla, scale bar, 100 μm. **(C)** Expression of *pdf* mRNA around the day in LD males (*n* = 3 independent repeats, error bar ± SEM). **(D)** PDF-immunostaining intensity over the day in weakly (weak) and strongly (strong) stained cells and dorsal protocerebrum arborizations (Dp) in LD males (*n* = 14–19 for each time point). Floating bars represent min to max values with the line at mean. **(E)** Expression of *pdf* in *cry-m* mutants (t-test (*) *p* < 0.05) (*n* = 3 independent repeats, error bar ± SEM). **(F)** PDF-immunostaining intensity in Dp arborizations in *cry-m* null mutants (Two-Way ANOVA genotype effect *p* < 0.0001, Tukey’s *post-hoc* (****) *p* < 0.0001, *n* for: WT = 18, *cry-m* ^*04−/−*^ = 27, *cry-m* ^*9in−/−*^ = 9). Floating bars represent min to max values with the line at mean. **(G)** Representative images of PDF-ir Dp arborization in WT and *cry-m* mutants. Grey rectangles on graphs depict darkness.

Using a monoclonal antibody against *Drosophila* PDF we mapped somata and neuronal projections containing PDF in the brain of *P. apterus* (Figure 1A). PDF immunoreactive (PDF-ir) cells were found exclusively in the optic lobes in the anterior-ventral base of the medulla. Two distinct types of PDF-ir cells are observed: four strongly stained cells (Figure 1B arrowhead) and two to four weakly stained cells (Figure 1B arrow). Based on the anatomical localization, which corresponds to PDF-ir cells in other Heteroptera (Vafopoulou et al., 2007; Ikeno et al., 2014; Hasebe et al., 2022) and species from other insects orders (Sehadova et al., 2003; Wei et al., 2010; Beer et al., 2018) they represent the group of lateral ventral neurons (LNv). Processes of these neurons form an extensive net of small ramifications in the crescent-like shape at the base of the medulla in the accessory medulla (aMe) region (Figure 1B). Weakly stained cells reside always at the base of aMe ramifications, while strongly stained cells are located more posteriorly and in several cases, we observed them as a separate group detached from aMe (Supplementary Figure S1C). Single PDF-ir fiber bundle runs along the medulla and arborizes heavily in the lamina. Additionally, one or two neuronal branches extending from the aMe innervate medulla (Figure 1B). LNvs send the neuronal process ventrally to the posterior part of the brain. From the main ventral PDF-ir fiber, two major processes extend towards the dorsal part of the brain, one anteriorly and one posteriorly, and the anterior branch further ramifies extensively in the dorsal protocerebrum (Dp) with numerous visible PDF-ir varicosities. The fibers from the anterior dorsal PDF path form most probably the bridge connecting contralateral hemispheres. Additionally, we observed the presence of two weaker stained processes budding ventrally from the main ventral PDF-ir fiber. One of them branches out near the point of the offshoot of the anterior dorsal fiber and runs in parallel to it towards the center of a brain (Figure 1A arrow). The second process branches out near the outgrowth of the posterior dorsal fiber and extends perpendicularly towards the posterior part of the brain (Figure 1A arrowhead). These two processes are observed in approximately half of the samples and in most cases are visible in only one hemisphere. If both processes are present in the brain they are located randomly in ipsilateral or contralateral hemisphere, therefore we do not assume that their absence is due to different staining in two halves of the brain. We were not able to track which cells give neuronal processes to the optic lobes and which innervate the dorsal protocerebrum.

### PDF Expression Is CRY-m Dependent

Analysis of *pdf* mRNA (Figure 1C) and PDF staining intensity in cells and neuronal processes in the dorsal protocerebrum in LD males heads (Figure 1D) does not indicate the cycling nature of the PDF transcript or protein (Kruskal-Wallis test *p* > 0.05, JTK_Cycle *p* > 0.05). It is worth mentioning that the staining intensity varies substantially between samples, even when samples originated from the same cohort of animals and were processed simultaneously. This huge variability could mask the presence of possible PDF cycling. Therefore, we cannot clearly define if the PDF levels change during the day in LD males. To check if PDF expression is clock dependent we analyzed the level of *pdf* transcript and protein level in described previously arrhythmic *P. apterus cry-m* mutants (*cry-m* ^*04*^ and c*ry-m* ^*9in*^) (Kotwica-Rolinska et al., 2021). Since we did not observe *pdf* changes during the day in WT, the expression was checked in samples collected only during the day (ZT8), and PDF staining intensity during the day and night (ZT8 and ZT20). In both *cry-m* mutant lines the level of *pdf* transcript (*t*-test *p* < 0.05) (Figure 1E) and the intensity of PDF-ir in the Dp is lowered (Figures 1F,G) (Two-way ANOVA, genotype effect test *p* < 0.0001, followed by Tukey’s test *p* < 0.0001). These results show that *pdf* expression and PDF release are CRY-m dependent.

### *pdf* Mutants Generated by CRISPR/Cas9

**FIGURE 2:**
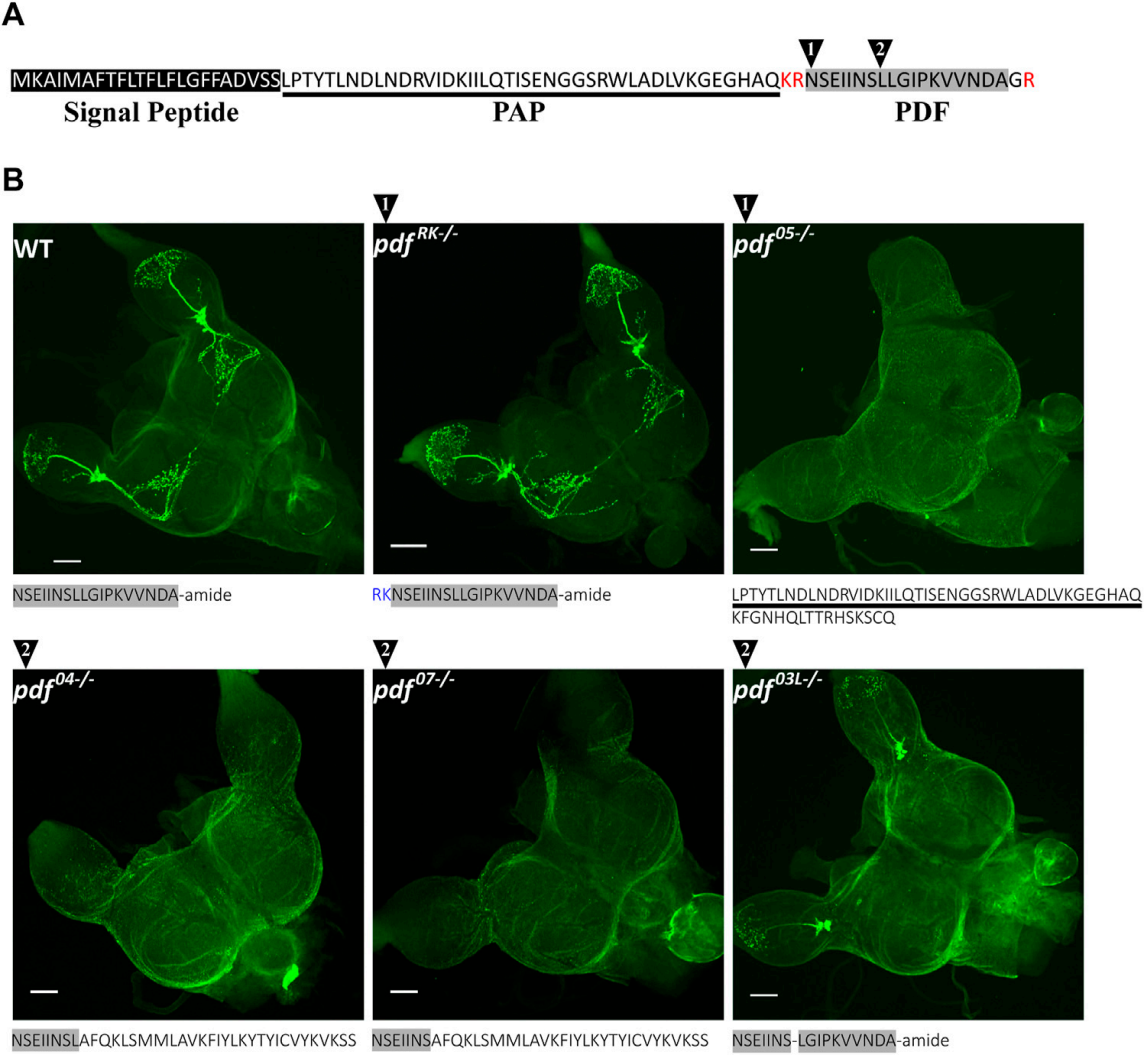
*P. apterus* PDF structure and CRISPR/Cas9 generated *pdf* mutants. **(A)** Peptide sequence of *P. apterus* PDF precursor consists of: signal peptide (black rectangle), PDF-associated peptide (PAP, underlined) and PDF active peptide (grey rectangle). PDF active peptide is flanked by basic amino acids predicted as a prohormone convertase cut sites (in red) and the last glycine is predicted to be amidated. Black triangles mark the position of the CRISPR/Cas9 target cut sites. **(B)** Amino acid sequences and PDF-immunostaining in WT and *pdf* mutants. Grey rectangle mark sequences identical to the original active PDF peptide. Note that *pdf* ^*RK−/−*^ mutant has an identical staining pattern to WT, whereas *pdf* ^*04−/−*^, *pdf* ^*05−/−*^, and *pdf* ^*07−/−*^ mutants result in no PDF immunoreactivity. In-frame deletion in *pdf* ^*03L−/−*^ removes conserved Leucine^8^ and produces altered staining pattern without Dp arborizations and extrusions to the Me. PDF-immunostaining intensity in La is weaker compared to WT.

PDF in *P. apterus*, similarly to other insects, is produced from the precursor which consists of the predicted signal peptide (Almagro Armenteros et al., 2019), PDF-associated Peptide (PAP) followed by the PDF active peptide (Figure 2A). *P. apterus* PDF is an18-mer peptide with predicted C-terminal amidation and has a conserved sequence similar to PDF in other insects species (reviewed in (Meelkop et al., 2011; Shafer and Yao, 2014). PDF sequence is flanked by the set of basic amino acids, which are predicted to be prohormone convertase cleavage sites in insects (Figure 2A) (Southey et al., 2006).

With the use of CRISPR/Cas9, we created mutants in the active PDF peptide region. For this purpose, two different guide RNAs were used. The position of the target region is marked in Figure 2A by black numbered triangles (for the detailed position in the genomic region and the nucleotide sequences of obtained mutants refer to Supplementary Figure S1A). As a result, we obtained five independent lines carrying unique mutations in the *pdf* gene: two lines with guide targeting region 1 and three lines in the target region 2. One of the lines in the region 1 (*pdf* ^*RK*^) carries 6bp nucleotide insertion, which results in the in-frame insertion of arginine and lysine downstream of the predicted cleavage site (WT cleavage probability of R67–0.8). These additional basic amino acids could serve as an alternative cleavage site for the prohormone convertase, leading eventually to the production of the wild-type PDF. However, Neuropred prediction of the mutated PDF prohormone shows that the cut site is more probably in the original position (cleavage probability of R67–0.8, R68–0.17, and K69–0.01) (Southey et al., 2006), suggesting that the new mutated peptide has additional RK amino acids inserted at the N-terminus. This mutation does not affect the PDF localization and the pattern of staining is identical as in WT (Figure 2B). However, the staining intensity is higher in *pdf* ^*RK*^ mutant than in WT, suggesting that the new mutated peptide could be more stable than WT PDF (Supplementary Figure S1B). Three of the mutants, one in region 1 (*pdf* ^*05*^) and two in the region 2 (*pdf* ^*04*^ and *pdf* ^*07*^) carry 5, 4, and 7 bp deletions respectively, causing a frame shift and change of the PDF sequence. Both *pdf* ^*04*^ and *pdf* ^*07*^possess the part of the original N-terminal PDF sequence (differ only by the presence or the lack of the conserved Leucine^8^), followed by a novel 29 long aa sequence. *pdf* ^*05*^ line has been predicted to have disrupted prohormone convertase cleavage site which leads to the production of the peptide consisting of the PAP peptide sequence with an additional 16 aa long novel sequence. These three mutants represent *pdf* null mutation which was confirmed by the lack of the PDF staining in the brain (Figure 2B). The last line (*pdf* ^*03L*^) carries 3bp deletion, which results in a WT-like PDF sequence lacking conserved Leucine^8^. This line shows that mutated PDF is still produced in the same number of cells as in WT, however, the intensity of staining in neuronal processes in the optic lobe is lowered, and disappears almost completely in the Dp arborizations and protrusions to the medulla region (Figure 2B and Supplementary Figure S1C).

### The Role of PDF in the Circadian Clock of *P. apterus*

**FIGURE 3:**
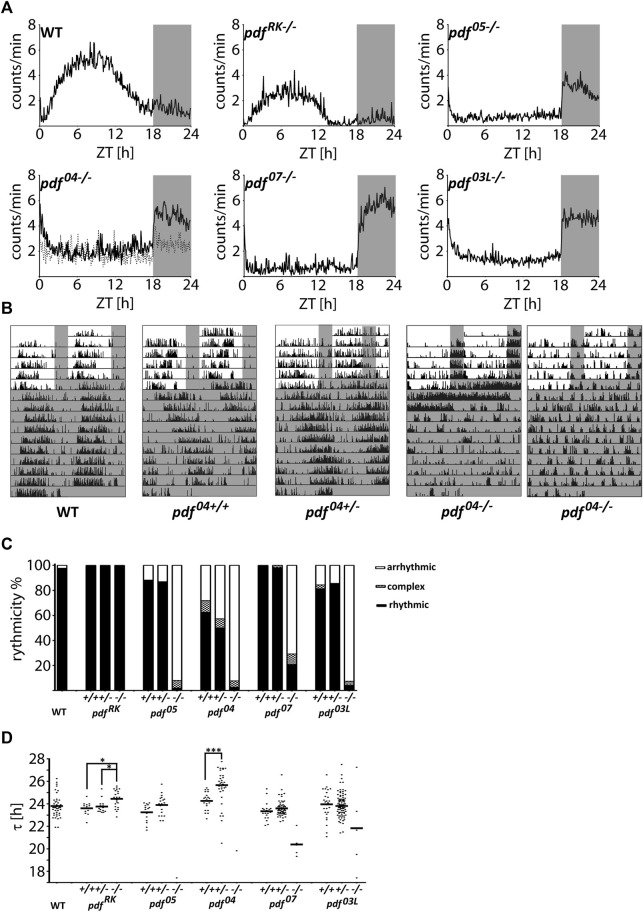
Locomotor activity of *P. apterus pdf* mutants. **(A)** The average daily activity of WT and *pdf* mutants in LD 18:6 cycles. Each graph represents the average activity of all bugs scored as “rhythmic in LD.” Dashed line in the graph representing *pdf* ^*04−/−*^ mutant shows the average activity of all bugs scored as “arrhythmic in LD.” **(B)** Representative actograms of WT, *pdf* ^*04+/+*^, *pdf* ^*04+/−*^, and, *pdf* ^*04−/−*^ bugs. Both types, nocturnal and arrhythmic in LD are shown in case of *pdf* ^*04−/−*^. Grey rectangles on graphs and actograms depict darkness. **(C)** Summary of the behavioral rhythmicity of WT and *pdf* mutants in DD. **(D)** Free-running period (τ) of rhythmic animals. Every dot represents a measurement from a single individual, the horizontal bar represents mean. Statistical differences are marked by (*) *p* < 0.05, (***) *p* < 0.001.

To assess the impact of PDF on the circadian clock function we analyzed the locomotor activity of *pdf* mutant animals. We observed two striking characteristics. Firstly, the majority (around 80%) of homozygous *pdf* null mutants (*pdf* ^*05−/−*^, *pdf* ^*04−/−*^ and *pdf* ^*07−/−*^) and *pdf* mutants lacking conserved Leucine^8^ (*pdf* ^*03L−/−*^) show predominantly nocturnal activity, while the remaining bugs are arrhythmic in LD (Figures 3A,B and Supplementary Table S2). Secondly, these mutants are almost completely arrhythmic in DD (Figure 3C and Supplementary Table S2), and in contrast to *Drosophila* (Renn et al., 1999), they lose rhythmicity instantly after the transfer to constant conditions (Figure 3B). The few remaining rhythmic bugs show very short τ, except *pdf* ^*03L−/−*^, whose τ spans between 17.42 and 27.25 h (Figure 3D and Supplementary Table S2). Both disrupted activities in LD and DD are recessive, since respective *pdf* ^*+/+*^ and *pdf* ^*+/−*^ retain WT phenotype. In contrast, *pdf* ^*RK−/−*^ mutant, which shows a similar staining pattern to the WT (Figure 2B), phenotypically also resembles the activity pattern of WT bugs in LD and DD (Figure 3A and Supplementary Table S2), but shows approximately 45 min longer τ than WT, *pdf* ^*RK+/+*^ or *pdf* ^*RK+/−*^ (Kruskal Wallis test, *p* < 0.01, Dunn’s *post-hoc p* < 0.01, *p* < 0.05 and *p* < 0.05, respectively) (Figure 3D and Supplementary Table S2). Significantly longer τ is also observed for *pdf* ^*04+/−*^ when compared to *pdf* ^*04+/+*^ (Mann-Whitney test *p* < 0.001).

### The Circadian Clock Gene Expression Is Disrupted in *pdf* Mutants but Is Not Responsible for the Nocturnal Behavior

**FIGURE 4:**
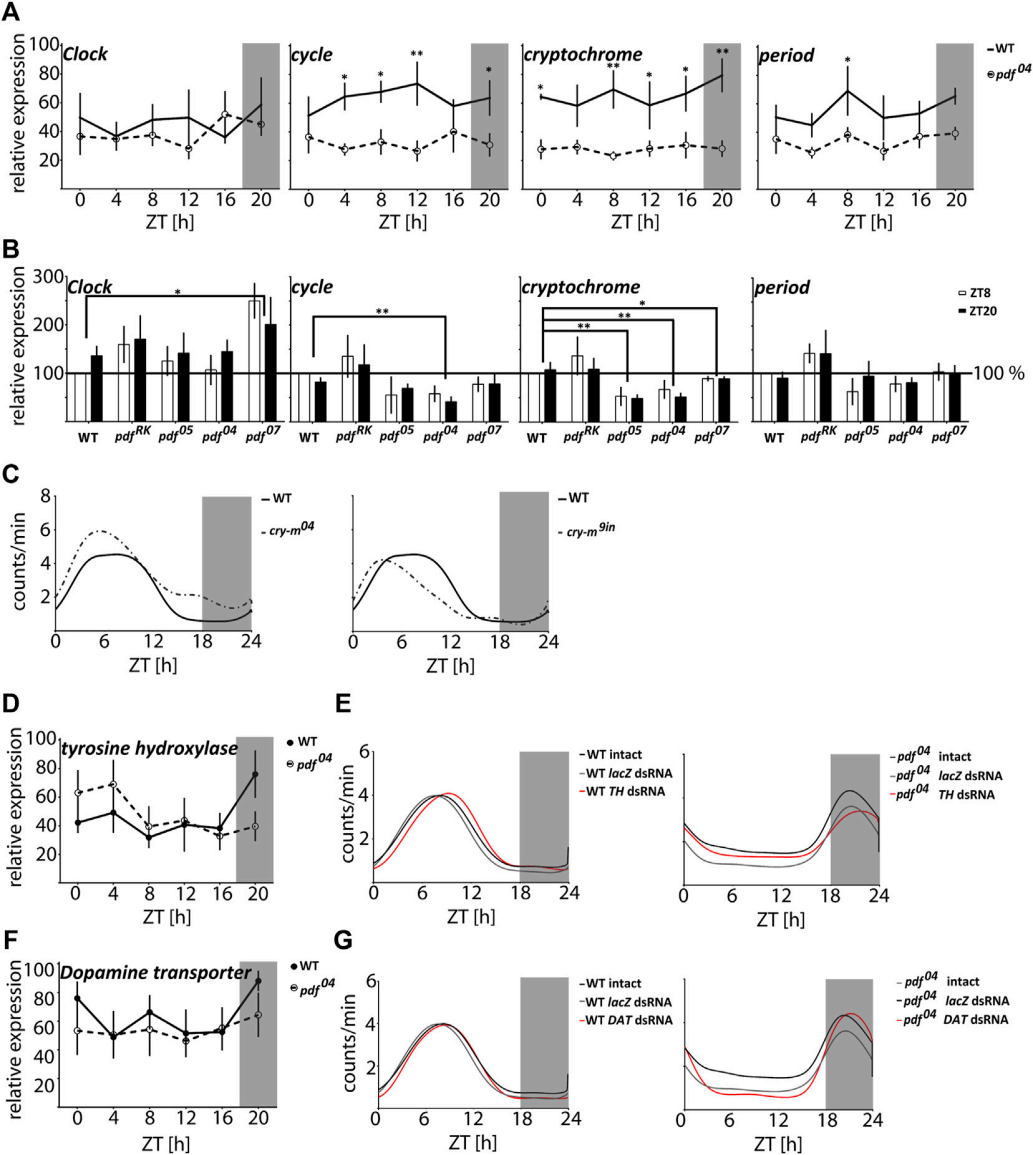
The nocturnal activity of *pdf* null mutants is independent of either the circadian clock or dopamine signaling. **(A)** Circadian clock gene expression in heads of WT (full circles, solid line) and *pdf* ^*04−/−*^ mutant males (empty circles, dashed line) throughout the day (*n* = 4 independent repeats, error bar ± SEM). **(B)** Circadian clock gene expression in heads of WT and *pdf* mutant males at ZT8 and ZT20 (*n* = 5 independent repeats, error bar ± SEM). Statistical differences are marked by (*) *p* < 0.05, (**) *p* < 0.01, (***) *p* < 0.001 **(C)** The smoothed average locomotor activity of *cry-m* ^*04−/−*^ and *cry-m* ^*9in−/−*^ males in LD 18:6 cycles. **(D)** Expression of tyrosine hydroxylase (TH) in heads of WT (full circles, solid line) and *pdf^04−/−^* mutant males (empty circles, dashed line) throughout the day (*n* = 4 independent repeats, error bar ± SEM). **(E)** The smoothed locomotor activity of intact, lacZ *lacZ* dsRNA and *TH* dsRNA treated WT (on the left) and *pdf^04−/−^* males (on the right). **(F)** Expression of Dopamine transporter (DAT) in heads of WT (full circles, solid line) and *pdf^04−/−^* mutant males (empty circles, dashed line) throughout the day (*n* = 4 independent repeats, error bar ± SEM). **(G)** The smoothed locomotor activity of intact, *lacZ* dsRNA and *DAT* dsRNA treated WT (on the left) and *pdf* ^*04−/−*^ males (on the right). Graphs represents the average activity of all bugs scored as “rhythmic in LD.” Grey rectangles on graphs depict darkness.

In order to analyze if the circadian clock is affected in *pdf* null mutants, we analyzed the daily changes of the core clock gene transcripts in WT and *pdf* ^*04--/*^. Surprisingly we did not detect the daily cycling in any of the genes tested in WT nor in *pdf* ^*04--/*^ (Kruskal-Wallis test *p* > 0.05, JTK_Cycle *p* > 0.05) but in the latter, expression of *cyc, cry-m,* and *per* was significantly downregulated independently of the time of the day (Two-way ANOVA time of the day effect *p* > 0.05, and genotype effect *p* < 0.0001, for *cyc* and *cry-m* and *p* < 0.005 for *per*) (Figure 4A). To prove that observed differences in clock genes expression are caused in fact by disruption of PDF we analyzed the same clock genes during the day and night in all predicted *pdf* null mutants (*pdf* ^*05−/−*^, *pdf* ^*04−/−*^ and *pdf* ^*07−/−*^) and as a control, we used the mutant showing WT phenotype *pdf* ^*RK−/−*^ (Figure 4B). Again we did not observe the effect of a time of a day on the circadian clock genes expression level. All *pdf* null lines show lowered expression of *cry-m* compared to WT (Mann Whitney test *pdf* ^*05−/−*^ *p* < 0.01 *pdf* ^*04−/−*^ *p* < 0.01, *pdf* ^*07−/−*^ *p* < 0.05). *cyc* is also lowered in all three lines, however, significantly only in *pdf* ^*04−/−*^ (Mann-Whitney test *p* < 0.01). The third null mutant, *pdf* ^*07−/−*^, on the other hand, shows a significant increase in *Clk* expression (Mann-Whitney test *p* < 0.01). Control line *pdf* ^*RK−/−*^ shows increased expression of all genes tested when compared to WT, however, none of these differences reached statistical significance (Mann-Whitney test *p* > 0.05). Our results show that the clock gene expression is strongly line-dependent, however mutation in *pdf* affects to some extent the function of the core circadian clock (Figure 4B). Since *pdf* null mutation affects particularly the expression of *cry-m* we wondered if this is the reason for the nocturnal activity of *pdf* mutants. To test this hypothesis, we analyzed daily activity in the *cry-m* ^*04−/−*^ (null) mutants and *cry-m* ^*9in−/−*^ (9aa insertion in the conserved CRY-m region). Both mutants with *cry-m* disruption showed loss of rhythmicity in constant conditions - as reported previously (Kotwica-Rolinska et al., 2021) but remained diurnal with the activity phase advanced to WT bugs (Figure 4C and Supplementary Table S2). Additionally, all of the bugs analyzed here showed clear rhythm in the daily behavior in contrast to *pdf* mutants, in which around 15–30% of bugs are active without preference to the time of a day (Supplementary Table S2). Our data show that the nocturnal behavior of *pdf* mutants is circadian clock independent.

### The Dopamine Signaling Disruption Is Not Responsible for the Nocturnal Activity of *pdf* Mutants

The nocturnal activity of normally crepuscular fruit flies is described for animals with an increased level of dopamine in the synaptic cleft in *Dopamine transporter* (*DAT*) mutants and by hyperactivation of the dopamine receptor (Lee et al., 2013). Additionally, the increased night-time activity of one of the circadian clock mutants *Drosophila Clk* ^*Jrk*^ (Allada et al., 1998) is also linked to the increased expression of the key enzyme in dopamine synthesis - *tyrosine hydroxylase* (*TH*) (Kumar et al., 2012). Therefore we analyzed if the nocturnal activity of *pdf* mutants could be also caused by the disruption in the dopamine signaling. The expression of *TH* and *DAT* does not cycle in *P. apterus* WT and *pdf* ^*04−/−*^ heads (Kruskal Wallis test *p* > 0.05, JTK_Cycle *p* > 0.05) and does not show a difference between these two genotypes (Figures 4D,F) (Two-way ANOVA *p* > 0.05). Moreover, downregulation of expression of *TH* or *DAT* by RNAi does not result in the nocturnal activity of WT bugs (Figures 4E,G) nor switches the activity of *pdf* ^*04−/−*^ to diurnal. The decrease of *TH* and *DAT* in WT and *pdf* ^*04−/−*^ has a subtle effect on the daily activity and disrupts slightly the robustness of rhythmicity in constant conditions in WT (Supplementary Figure S3 and Supplementary Table S2). *TH* dsRNA causes a significant 1 h delay of the activity onset and offset compared to the intact WT and WT treated with *lacZ* dsRNA (Watson’s U^2^ test *p* < 0.05) (Figure 4E), while *DAT* dsRNA delays only the onset of activity by approximately 30 min (Watson’s U^2^ test *p* < 0.1) (Figure 4G). In *pdf* ^*04−/−*^ *TH* and *DAT* dsRNA do not show a significant effect on the phase of the activity onset and offset. All analyzed data show circular distribution (Rayleigh test *p* < 0.0001, rho 0.79–0.98). Taken together our data show that dopamine signaling is involved in the fine-tuning of the function of the circadian clock in *P. apterus* but do not support the hypothesis that the nocturnal activity of *pdf* mutants is caused by dopamine signaling disruption.

### Response of *pdf* mutants to the Light and Darkness Is a Transient Masking Effect

**FIGURE 5:**
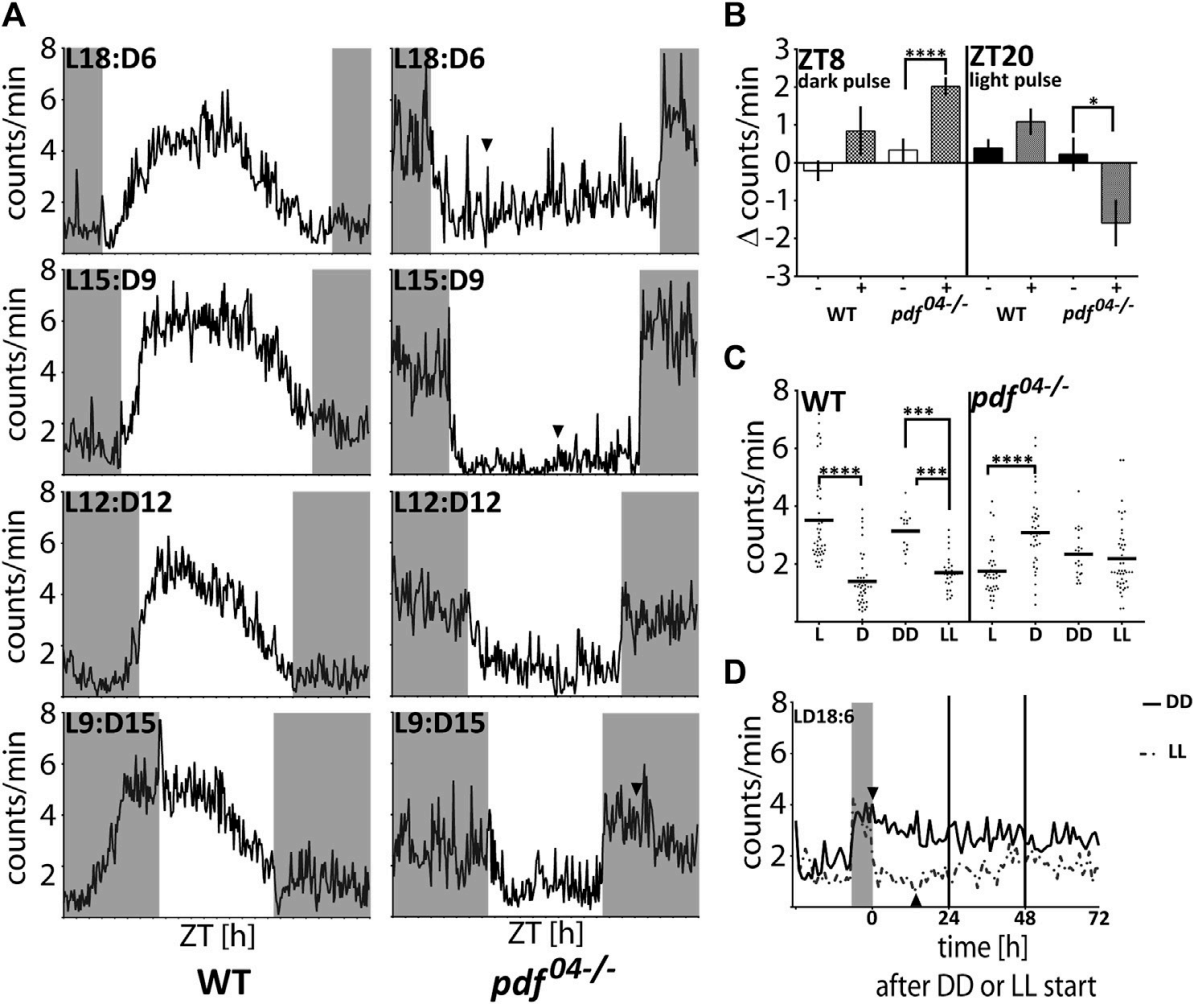
The response of WT and *pdf* ^*04−/−*^mutants to light and darkness. **(A)** The average locomotor activity of WT and *pdf* ^*04−/−*^ in different photoperiodic regimes. In *pdf* ^*04−/−*^ mutants in photoregimes L18:D6 and L15:D9, the activity during the light phase starts to increase several hours after the initial drop (arrowhead). In the photoregime L9:D15, the activity of *pdf* ^*04−/−*^ starts to fall several hours after the initial spike at the beginning of the night (arrowhead). Activity graphs represents the average activity of all bugs scored as “rhythmic in LD.” Grey rectangles on graphs depict darkness. **(B)** The effect of “dark pulses” applied at ZT8 and “light pulses” applied at ZT20 on the average activity of WT (*n* = 32, *n* = 35, respectively) and *pdf* ^*04−/−*^mutants males (*n* = 59, *n* = 39, respectively). **(C)** Average activity of WT and *pdf* ^*04−/−*^ during the light (L) and darkness (D) in LD18:6 cycles, constant darkness (DD) and constant light (LL). Every dot represents a measurement from a single individual. In LD cycles the activity during the light or darkness was averaged for three last days. In DD and LL activity was averaged from the total number of minutes during 10 days of measurement. The horizontal bar represents mean. Statistical differences are marked by (***) *p* < 0.001, (****) *p* < 0.0001. **(D)** The average activity of *pdf* ^*04−/−*^ mutants on the last day in LD 18:6 and three following days in constant darkness (DD - solid line) or constant light (LL-dashed line). The level of the activity becomes comparable on the third day in both constant conditions. Activity graphs represents the average activity of all bugs used in the experiment. The grey rectangle on the graph depicts the last night time in the LD cycle. Arrowheads point to the time when the activity starts to decrease in DD and starts to increase in LL.

In order to investigate further the cause of the nocturnal activity of *pdf* mutant bugs, we performed a set of analyses by applying different light:dark schedules. First, we analyzed if *pdf* null bugs are able to adjust their activity to different photoperiods. On graphs, the photoperiod is marked by Lx:Dy – which describes xh of light and yh of darkness (Figure 5A). While WT bugs are predominantly diurnal in all photoperiods, most *pdf* mutants start to be active at the beginning of the night and cease to be active at the beginning of the day, irrespectively of the photoperiod tested (Figure 5A). Both, WT and *pdf* ^*04−/−*^ adjust to the new photo-regime immediately, already on the next day after the transfer to the new conditions (not shown). Interestingly, WT bugs activity offset occurs always before the light-off, while activity onset does not adjust to the new time of the light-on in shorter photoperiods (Figure 5A). In contrast, in *pdf* mutants, the onset of activity occurs concomitantly with the light-off, and activity offset occurs shortly after the light-on. These data suggest that the nocturnal activity is possibly a passive response to the change in the light conditions. To address this further we checked the response of WT and *pdf* ^*04−/−*^ mutants to the 2 h pulses of darkness applied in the middle of the day (ZT8) and 2 h light pulses applied in the middle of the night (ZT20). The activity of WT bugs increased slightly after both types of pulses. In contrast, *pdf* mutants significantly increased their activity during the dark pulse (Mann Whitney test *p* < 0.0001) and suppressed substantially their activity during the light pulse (Mann Whitney test *p* < 0.05) (Figure 5B). To check which conditions - light or darkness could have a stronger effect on the *pdf* mutant’s activity we analyzed the level of their activity in LD18:6 cycles, constant darkness (DD), and constant light (LL) conditions. In LD18:6, WT bugs show significantly higher activity during the light (L) and *pdf* ^*04−/−*^ during the darkness (D) (Figure 5C) (Mann-Whitney test *p* < 0.0001), as expected. WT bugs are rhythmic in DD and 60% retain rhythmic behavior in LL (Supplementary Table S2), as described previously (Kaniewska et al., 2020). Surprisingly, the average activity in DD and LL is comparable in *pdf* ^*04−/−*^ mutants (Figure 5C) (Mann-Whitney test *p* > 0.05). Additional analysis of the activity in initial days in DD and LL conditions showed that the raise of the activity in the darkness and its drop in the light lasts approximately 4–6 h and is followed by slow restoration to the intermediate level (Figure 5D). A similar situation can be noticed when bugs experience very short or long photoperiods (Figure 5A arrowheads). Taken together our data show that nocturnal activity in *pdf* mutants is caused by clock independent transient masking effect.

### PDF Is Involved in the Photoperiod-dependent Reproductive Diapause

**FIGURE 6:**
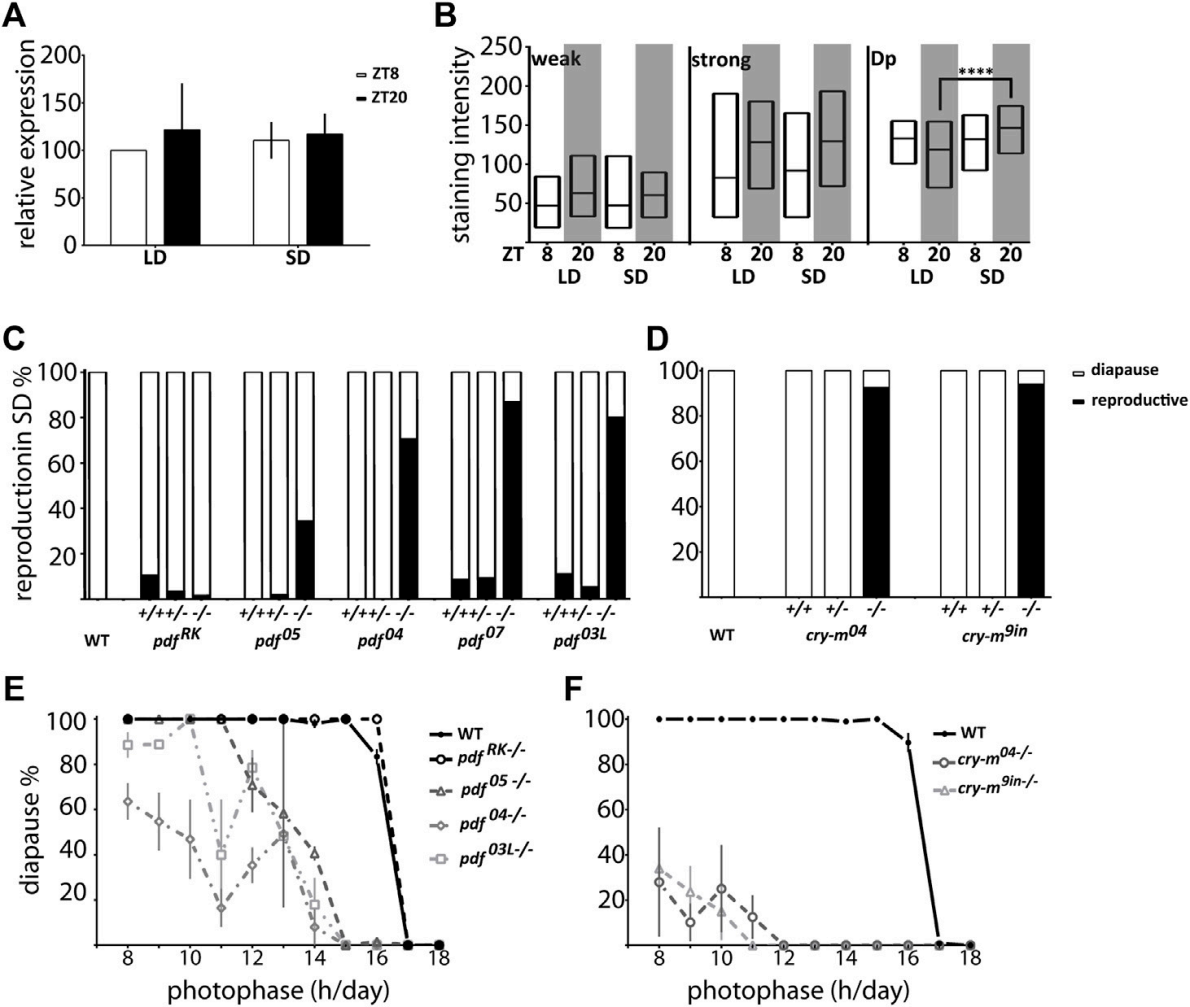
Connection of *P. apterus* PDF and CRY-m to the photoperiodic response. **(A)** *pdf* expression in female heads during the day and night in LD and SD (*n* = 3 independent repeats, error bar ± SEM). **(B)** PDF-immunostaining intensity in weakly (weak) and strongly (strong) stained cells and dorsal protocerebrum arborizations (Dp) in female brains during the day and night in LD and SD (*n* = 12–16, Kruskal-Wallis test: weak *p* > 0.05, strong *p* > 0.05, Dp *p* < 0.05 and Dunn’s *post hoc p* < 0.0001) Floating bars represent min to max values with the line at mean. Statistical difference is marked by (****) *p* < 0.0001. **(C)** Reproductive status of WT and *pdf* ^*+/+*^ *,* ^*+/−*^ *and* ^*−/−*^ in SD conditions. **(D)** Reproductive status of WT and *cry-m* ^*+/+*^, ^*+/−*^ *and* ^*−/−*^ in SD conditions. **(E)** The photoperiodic curve of WT and *pdf* homozygous mutants **(F)** The photoperiodic curve of WT and *cry-m* homozygous mutants Data presented as mean values with error bars ± SEM.

To explore if PDF is involved in the photoperiodic response we checked the reproductive status of *pdf* mutant females kept in diapause-inducing short-day photoperiod (SD 12:12). Our results show that while *pdf* ^*+/+*^ and *pdf* ^*+/−*^ are mostly diapausing in SD, most of *pdf* null homozygous mutant females are reproductive. This situation is also true for *pdf* ^*03L*^, but not the *pdf* ^*RK*^ mutants (Figure 6C), showing that the functional PDF is necessary for diapause induction under short-day conditions. These results point toward PDF as a diapause-inducing factor in *P. apterus*. Therefore, we expected that in diapause inducing conditions we will observe an increased level of PDF. Indeed, we observed increased staining intensity in the PDF arborization in the Dp during SD night, but not in the staining of PDF-ir cells (Figure 6B) and no photoperiod-dependent difference in the *pdf* transcript level (Figure 6A).

Since the level of PDF in the Dp arborizations is CRY dependent we asked if the *cry-m* mutants have similarly to *pdf* mutants disrupted reproductive arrest in SD conditions. Indeed, virtually all of *cry-m* homozygous mutants are reproductive in SD (Figure 6D). Interestingly, in contrast to *cry-m*, a substantial number of *pdf* null mutants is still able to induce diapause in SD (Figure 6C), therefore we asked if PDF is absolutely necessary for the photoperiodic timer mechanism. To answer this we analyzed the reproduction of females kept under different photoperiods ranging from LD 18:6 to LD 8:16 (Figure 6E). Control WT animals show clear photoperiodic response, as described previously (Saunders, 1983), with the critical day-length (CDL) 16.41 h (S.D. ± 0.10). *pdf* ^*RK−/−*^ mutants show a similar photoperiodic curve to WT but the CDL is approximately 20 min longer (CDL = 16.60h, S.D. ± 0.13). The response of other mutants tested is line dependent. *pdf* ^*05−/−*^ become completely diapausing in LD 11:13 (CDL 13.58h, S.D. ± 0.29), *pdf* ^*03L−/−*^ bugs reach the 100% of diapause in LD 10:14 (CDL 13.49h, S.D. ± 0.42) but *pdf* ^*04−/−*^ are reproductive in a wide range of photoperiods and they never show 100% of reproductive arrest (predicted CDL 13.70h, S.D. ± 0.41) (Figure 6E). Nevertheless, the percentage of incidence of diapause in these *pdf* mutant lines grows with the shortening of the photoperiod, which implies that the photoperiodic response is modulated but not destroyed by the lack of functional PDF. If PDF - a key output of the circadian clock - is important but not necessary for the photoperiodic response we wondered if the functional circadian clock is necessary for the photoperiod measurement. Therefore, we analyzed the response to the photoperiod of *cry-m* mutants. Bugs with disrupted CRY-m are predominantly reproductive, and even though some bugs are still able to induce reproductive arrest, especially in shorter photoperiods, the percentage of *cry-m* diapausing bugs is much smaller than in the case of PDF-depleted bugs. Therefore, it seems that CRY-m is necessary for the proper photoperiodic response. Additionally, our data suggest that other circadian clock-dependent or just *cry-m* dependent factors act synergistically with PDF in transmitting the information about the day length to the photoperiodic timer.

## Discussion

PDF is one of the most studied neuropeptides in invertebrates. In Crustacea homologous peptide PDH regulates dispersing of the hormone in the epidermis and light-dependent pigment movement in the retina (Meelkop et al., 2011) and in insects, PDF plays a role as an output of the circadian clock (Renn et al., 1999). Injection of the PDF shifts the phase of the activity of the clock in crickets (Singaravel et al., 2003), cockroaches (Petri and Stengl, 1997), and honeybees (Beer et al., 2018). Transcript downregulation by RNAi disrupts rhythms in crickets (Hassaneen et al., 2011) and cockroaches (Lee et al., 2009), and in *Drosophila pdf* (Renn et al., 1999) and *pdfR* mutants (Hyun et al., 2005; Lear et al., 2005; Mertens et al., 2005) gradually become arrhythmic in constant darkness. Most of the studies concerning the function of the PDF come from *Drosophila* due to the access to an amazing spectrum of genetic tools currently not available for other insect species, which makes conclusions about PDF action *Drosophila* biased. Here we present the first data describing PDF in the linden bug *P. apterus* (Hemiptera) and its role in the circadian and photoperiodic clocks in this species based on data obtained from CRISPR/Cas9 generated mutants.

### *P. apterus* PDF Localization

*P. apterus* PDF is produced in two distinct classes of neurons which are located at the base of the medulla and based on the similarity to the location in other insects they represent lateral neurons (LN) (Helfrich-Forster, 1995; Sehadova et al., 2003; Vafopoulou et al., 2007; Ikeno et al., 2014; Beer et al., 2018; Hasebe et al., 2022). These two groups of neurons differ by the strength of the PDF staining and the location of the cells. Weakly stained cells lay exclusively within the PDF arborizations in the region of the accessory medulla while strongly stained cells are located more posteriorly and are often more dispersed (Supplementary Figure S1C). This location and intensity of staining suggest that they could represent small and large lateral neurons described in *Drosophila* (Helfrich-Forster, 1995), however, we were not able to tract their projections which could give a closer answer if they really resemble the *Drosophila* s- and l-LNvs. In the optic lobe, these neurons ramify densely in the accessory medulla and lamina where based on the presence of PDF positive varicosities we expect that PDF is released and feedback on the clock and function of the visual system, respectively. PDF-ir cells projected their axons towards the dorsal protocerebrum where they ramify to the anterior and posterior parallel branches with the anterior branch showing intense staining of numerous varicosities close to the surface of the brain. The staining of PDF in *P. apterus* generally resembles PDF localization in other Heteroptera, however, *R. pedestris* (Ikeno et al., 2014), *Rhodnius prolixus* (Vafopoulou et al., 2007) and *P. stali* (Hasebe et al., 2022) lack the division of projections in the dorsal protocerebrum into anterior and posterior parallel branches.

### The Connection of the PDF to the *P. apterus* Circadian Clock

We were not able to state if the rhythm of the *pdf* expression and PDF level in PDF-ir cells and Dp arborizations exists in LD reared males. While we noticed a trend towards higher PDF levels during the night in the Dp region, substantial variability between samples prevents us to draw a definite a conclusion about the nature of PDF cycling. Interestingly, we also do not notice the cycling expression of any of the circadian clock genes, which is the known signature of the properly-functional circadian clock in *Drosophila* and other insect species. Similarly, lack of PDF cycling and non-rhythmic expression of the circadian clock genes was reported in another heteropteran species *R. pedestris* (Ikeno et al., 2008; Ikeno et al., 2014). We assume that, at least in *P. apterus*, the lack of cyclic expression could be the result of cycling occurring only in a few circadian clock cells and could also arise from the interspecies variability, since our previous results showed that the downregulation of the several circadian clock genes has the effect consistent with the typical transcriptional-translational feedback loop model (Kotwica-Rolinska et al., 2021). While we cannot state the presence or the absence of the rhythmicity of the PDF level, its expression and accumulation in the dorsal protocerebrum are CRY-m dependent, suggesting that PDF transcription and post-translational modifications could be possibly under control of other circadian clock genes in *P. apterus*. Non-rhythmic (Park and Hall, 1998) but circadian clock-dependent expression of the *pdf* is also reported for *Drosophila* and the cycling of PDF in s-LNvs axon terminals is absent in *per* and *tim* mutant flies (Park et al., 2000). Currently, we do not know if other circadian clock genes affect *P.apterus pdf* transcription or PDF accumulation, and how exactly CRY-m activates *pdf* expression leading to the increased PDF level. We assume that CRY-m can act indirectly *via* the regulation of the circadian clock machinery or affecting other pathways involved in the regulation of translation or neuropeptides release. If PDF in *P.apterus* is truly under the circadian clock control, we would expect that the downregulation of *per* should have a similar effect to CRY-m depletion, and the downregulation of the positive elements like *Clk* and *cyc* should up-regulate *pdf* expression and the level of PDF.

*P. apterus* PDF feeds back on the function of the circadian clock, since the expression of *cry-m* is downregulated in *pdf* null mutants. In *Drosophila*, PDF affects the machinery of the clock by stabilizing PER/TIM heterodimer (Seluzicki et al., 2014) and stimulating the CLK/CYC-dependent transcription in a time-dependent manner (Sabado et al., 2017). Whether PDF acts similarly on the *P. apterus* circadian clock remains to be addressed.

What is the functional role of the *P. apterus* PDF in the circadian clock? In *Drosophila*, *pdf* null flies lose the morning anticipation peak, display an advanced evening peak of activity, and become gradually arrhythmic in constant darkness (Renn et al., 1999), due to progressive asynchrony between circadian clocks centers in the brain (Lin et al., 2004). The *P. apterus pdf* mutants behave strikingly differently. The majority of them become instantly arrhythmic in constant conditions, while under LD conditions around 70–80% of them show nocturnal activity pattern, and the rest is arrhythmic. The instant loss of rhythmicity in the absence of PDF proves that in *P. apterus* PDF is an element of the functional circadian clock and serves as the main output pathway regulating behavioral rhythmicity. A similar hypothesis was drawn based on results obtained in the cockroach *Blattella germanica* where *pdf* RNAi increased arrhythmicity of animals in both LD and DD (Lee et al., 2009). The complete loss of PDF staining in *pdf* null mutants does not allow us to determine which PDF neuronal projections are responsible for the regulation of rhythmicity. Without an additional marker, we cannot exclude the possibility that PDF cells and neuronal projections do not develop normally in *pdf* null mutants. However, *pdf* ^*03L−/−*^ line lacking conserved Leucine^8^, lacks PDF-ir dorsal protocerebrum arborizations and shows circadian phenotype identical to *pdf* null mutants. Therefore, we think, that similarly to s-LNv arborization in *Drosophila* (Helfrich-Forster et al., 2007), dorsal protocerebrum arborizations could also be responsible for maintaining the rhythmicity in bugs. Interestingly, in *pdf* ^*03L−/−*^ both weak and strong PDF-ir cells are present, and aMe and lamina arborizations produce weaker staining (Supplementary Figure S1C). This selective disappearance of PDF could imply that PDF can be differently modified by post-translational mechanisms in different neuronal processes. In fact, two forms of PDF resulting from different posttranslational modifications are known in *Drosophila*, l-LNv produce amidated PDF, while s-LNv PDF is not amidated (Park et al., 2008). Our hypothesis assumes that except amidation, also the deactivation of PDF could be different in the optic lobe and Dp arborizations, leading to the production of peptides with different activity and stability. What could be a mechanism of the lower stability of the PDF in *pdf* ^*03L−/−*^? Incidentally, *Drosophila* PDF is inactivated by the synaptic membrane endopeptidase neprilysin, by cleavage between conserved Serine^7^ - Leucine^8^ (Isaac et al., 2007). We hypothesize that neprilysin could act predominantly in the dorsal PDF neuronal processes. In our scenario when Leucine^8^ is removed in *pdf* ^*03L−/−*^, the next Leucine^9^ takes its place, keeping the original cleavage site intact. If then, the mutated form is less resistant to proteolysis, the increased cleavage would lead eventually to almost complete loss of PDF in the dorsal protocerebrum. We are not able to identify whether this mutated PDF peptide, albeit still present in optic lobes, is functional.

The more intriguing aspect of *pdf* null mutants’ behavior is their partial arrhythmicity and predominant nocturnal activity in LD cycles. The role of PDF in setting up the preference time of locomotor activity was observed also in the cricket *G. bimaculatus*, where the typical nocturnal activity of nymphs was switched to diurnal after *pdf* RNAi. Moreover, these crickets adjusted faster to the new photoregime, placing PDF as a factor involved in the circadian clock entrainment (Hassaneen et al., 2011). In addition, PDF is predicted to be responsible for the preference of the timing of activity in artificially selected diurnal and nocturnal populations of *Drosophila* (Pegoraro et al., 2020). However, the actual link between PDF and a particular time of activity is missing. Here we show that in *P. apterus* the nocturnal activity of *pdf* mutants is clock independent since *cry-m* mutants are diurnal and show robust rhythmicity in LD conditions. Additionally, our earlier experiments using RNAi to downregulate the expression of several circadian clock genes in *P. apterus* did not affect bugs’ diurnal activity patterns (Kotwica-Rolinska et al., 2021). Next, we analyzed if the *pdf* nocturnal activity is a result of disrupted dopamine signaling since similar night-time activity was reported for *Drosophila* with an increased level of dopamine signaling in: *1*) flies treated with the dopamine D2 Receptor agonist *2*) mutants with disrupted dopamine transporter (Lee et al., 2013) and *3*) *Clk* ^*Jrk*^ mutants with an increased expression of *tyrosine hydroxylase* – the rate-limiting enzyme important for dopamine precursor (Kumar et al., 2012). However, our data suggest that the disruption of dopamine signaling is not the main reason for the nocturnal activity observed in *pdf* mutants, since the expression level of *TH* and *DAT* is comparable between WT and *pdf* null mutants and downregulation of either of enzymes does not change the time of the activity in WT and *pdf* ^*04−/−*^ bugs. However, we cannot completely exclude the possibility that the targeted transcripts depletion, while quite efficient (Supplementary Figure S2A), could be still insufficient to successfully disrupt dopamine signaling.

*P. apterus pdf* mutants, in contrast to *Drosophila* (Yoshii et al., 2009) immediately adapt to new photoperiods always keeping the night-active mode. Additionally, light pulses during the night and dark pulses during the day elicit an immediate and opposite change of behavior, suggesting this response is a passive masking effect. However, the light suppresses and darkness increases the level of the activity only in a transient manner. The nocturnal activity of artificially selected *Drosophila* is also a form of masking effect to LD cycles and flies released to DD exhibit activity with the phase matching “diurnal-like” activity (Pegoraro et al., 2020). While we still don’t know the reason of the *pdf* null mutants’ nocturnal behavior, we assume that the PDF role could be connected with the light adaptation mechanism. PDF shows rich innervation in the lamina, which could impact the function of photoreceptor cells, or the action of the lamina interneurons involved in the light transmission. Involvement of PDF was already proven for screening pigment migration in the photoreceptors (Pyza and Meinertzhagen, 1998) and daily changes of axon size of lamina L1 and L2 visual interneurons in *Musca domestica* (Pyza and Meinertzhagen, 1996). However, the exact mechanism of the PDF action on the visual system in *P. apterus* needs to be elucidated.

### Connection of the CRY-m and PDF to the photoperiodic Regulation of the Reproductive Diapause

The involvement of the clock in the photoperiodic timer is still under debate. However, genetic manipulation of the circadian clock genes by the use of RNAi or knock-out mutants proved their impact on diapause induction in butterflies *D. plexippus* (Iiams et al., 2019) and *Bombyx mori* (Ikeda et al., 2021), mosquito *C. pipens* (Meuti et al., 2015), cricket *Modicogryllus siamensis* (Sakamoto et al., 2009) as well as true bugs *R. pedestris* (Ikeno et al., 2010; Ikeno et al., 2011) and *P. apterus* (Kotwica-Rolinska et al., 2017). Only few studies focused on the role of the circadian output neuropeptide PDF in the photoperiodic response. PDF was shown to have impact on the reproductive status in *Drosophila* and *C. pipens*, where it plays a role as a reproduction-inducing factor. Contrary, the recent report shows that in the stink bug *P. stali* downregulation of *pdf* by RNAi promotes reproduction in SD putting PDF as a diapause promoting factor (Hasebe et al., 2022), while in *R. pedestris* RNAi did not affect the reproductive status of the bugs (Ikeno et al., 2014). In the same study surgical removal of the region containing PDF cells led to female reproduction under SD conditions suggesting that other factors released by the same cells may be responsible for diapause induction.

In our study, we show that CRY-m and PDF are involved in the photoperiodic regulation of reproductive diapause in *P. apterus*. Similarly to *R. pedestris* (Ikeno et al., 2011) and *P. stali* (Hasebe et al., 2022), disruption of either *cry-m* or *pdf* inhibits reproductive arrest in SD in *P. apterus*. Based on these results we conclude that PDF functions as a diapause-promoting factor in *P. apterus*. Diapause status of WT females coincides with the increased level of PDF in the dorsal protocerebrum during the night but not during the day. Additionally, the *pdf* mRNA level and PDF level in cell somata are photoperiod-independent. What is then a possible explanation of the photoperiodic regulation of PDF level in Dp and how could these changes be connected to the diapause induction? We hypothesize that PDF in Dp could be accumulated during the darkness and released during the day. This would explain the higher PDF level in Dp which we observe at ZT20 (but not at ZT8) in SD. In SD at ZT20, bugs already experienced 8 h of darkness (light-off is at ZT12) and in LD 2 h of darkness (light-off is at ZT18), while in both conditions, at ZT8 bugs experienced the same duration of the light (8 h). The hypothesis which assumes prolonged PDF accumulation due to longer nights in SD, could also explain the increased level of PDF in Dp without photoperiod-dependent differences in the mRNA or PDF level in cells somata. Additionally, longer accumulation of PDF during the night in SD conditions would lead to the increased level of available PDF, whose later increased release could be a signal for the diapause induction. However, this hypothesis needs to be further addressed by analyzing additional time points and analyzing the PDF level in bugs kept in different photo-regimes.

The hypothesis that the increased level of PDF signals diapause induction in *P. apterus* could also explain the predominantly reproductive status of *cry-m* mutants, whose PDF level in the dorsal protocerebrum is decreased. Additionally these data suggest, that the response to the photoperiod is mediated by the CRY-m dependent regulation of the PDF level. Interestingly, not all *pdf* null mutants but almost all *cry-m* mutants are reproductive in SD. Furthermore, while the majority of *cry-m* mutants stay reproductive even in shorter photoperiods, the disruption of the PDF, the CRY-m dependent key output of the circadian clock, did not make bugs completely unresponsive to the photoperiodic changes. The incidence of diapause increases with the shortening of the light phase and in the case of *pdf* ^*05*^ and *pdf* ^*03L*^ shorter photoperiod leads all females to arrest their reproduction. Nevertheless, we show that the lack of PDF shortens the CDL in the photoperiodic diapause induction. The CDL is a variable trait and depends on many factors, including the temperature, food availability, geographical origin of the species (reviewed in (Denlinger et al., 2017), and is most probably under polygenic control. To our knowledge, this is the first report showing the direct involvement of the single neuropeptide - *pdf* - in the modulation of the complex trait like CDL. PDF was shown to be connected to the behavioral adaptation to life in different latitudes in several *Drosophila* species (Menegazzi et al., 2017; Bertolini et al., 2019), and probably could be also responsible for differences observed in latitude-depedent photoperiodic responses. Interestingly, recent data suggest that PDF action can have an even more complex nature, and that PDF could be involved as a molecule marking the change in the photoperiod bi-directionally (Hasebe et al., 2022). In *P.stali*, *pdf* RNAi inhibited ovarian arrest after transfer from reproducing to diapause-inducing conditions. On the other hand, the downregulation of *pdf* also prolonged the time of the oocyte maturation after the transfer from SD to LD conditions. It needs to be elucidated if PDF can play a similar role in *P.apterus*.

Even though the photoperiodic response is clearly modified in *P. apterus pdf* mutants, these bugs can still initiate diapause program in shorter photoperiods. These results suggest there are other important circadian clock-dependent, or at least CRY-m regulated factors involved in the photoperiodic response in *P. apterus*. A similar conclusion was drawn for *R. pedestris* where *pdf* RNAi did not affect diapause, while removing part of the brain containing PDF cells induced reproduction under SD conditions (Ikeno et al., 2014). Moreover, it was proven that in *Drosophila* sNPF acts synergistically with PDF to inhibit reproductive arrest *via* activation of insulins producing cells in the brain (Nagy et al., 2019). Additionally, other neuropeptides, like diuretic hormone 44 (DH44) (Hasebe and Shiga, 2021) or Myoinhibitory peptides (MIPs) (Tamai et al., 2019) whose release is under circadian clock control, were shown to also participate in the regulation of imaginal diapause. Taken together, our data show that the photoperiodic regulation of the *P. apterus* diapause is regulated by CRY-m *via* the regulation of the PDF level in the Dp and others, currently unknown factors.

Interestingly, as mentioned before, some of the *cry-m* mutants still show the ability to diapause in the shorter photoperiods and retain rudimentary photoperiodic response. Analogous results were obtained for arrhythmic *Drosophila per* null mutants which, similarly to WT flies, were able to induce ovarian arrest at the low temperature and short photoperiods but their CDL was 2 h shorter than in WT flies (Saunders et al., 1989). The authors proposed three explanations of these results: *1*) the circadian clock is not involved in the photoperiod measurement; *2*) the circadian clock is involved but *per* is not essential; *3*) the clock still functions to some level in *per* ^*0*^ flies, which show residual non-circadian rhythmicity and ability to entrain to LD cycles. While Saunders et al. (1989) favored the second hypothesis, we assume, that the presence of the weak photoperiodic response in *P. apterus cry-m* mutants’ females could appear in individuals, which retained some of the circadian clock function. In support of our hypothesis, we observed that a substantial proportion of *cry-m* mutant males were rhythmic, albeit their rhythmicity was considered as complex, that is multiple non-24 h components were detected (Kotwica-Rolinska et al., 2021). Interestingly, similar aberrant residual rhythmicity was reported for *per* ^*01*^ *Drosophila* mutants, where about 30% of flies displayed a non-24-h periodicity (Helfrich-Förster, 2001). Thus, even the third hypothesis might be considered for the results of Saunders et al. (1989).

## Conclusion

In our study, we present the circadian clock and diapause phenotypes of several lines of *pdf* and *cry-m* mutants created with the CRISPR/Cas9 technology. Our results show that PDF is regulated by CRY-m and possibly is the only output factor from the circadian clock synchronized by the light: dark cycles. PDF independently of the circadian clock regulates acute response to the light and probably plays a role in light:dark adaptation. CRY-m and PDF are diapause-inducing factors under SD conditions. The *cry-m* mutants lose almost completely the ability to diapause while *pdf* mutants retain the ability to respond to the photoperiod pointing to the existence of some additional factors which act downstream of the CRY-m in the mechanism of clock dependent diapause induction.

## Data Availability

The original contributions presented in the study are included in the article/Supplementary Material, further inquiries can be directed to the corresponding author.
