# Supplementary material for: Pigment Dispersing Factor Is a Circadian Clock Output and Regulates Photoperiodic Response in the Linden Bug, Pyrrhocoris apterus
Source: Front Physiol. 2022 Apr 29;13:884909. doi: 10.3389/fphys.2022.884909 (PMC9099023; doi:10.3389/fphys.2022.884909)
Supplement: Supplementary file 1 [file DataSheet2.pdf]

Supplementary Material

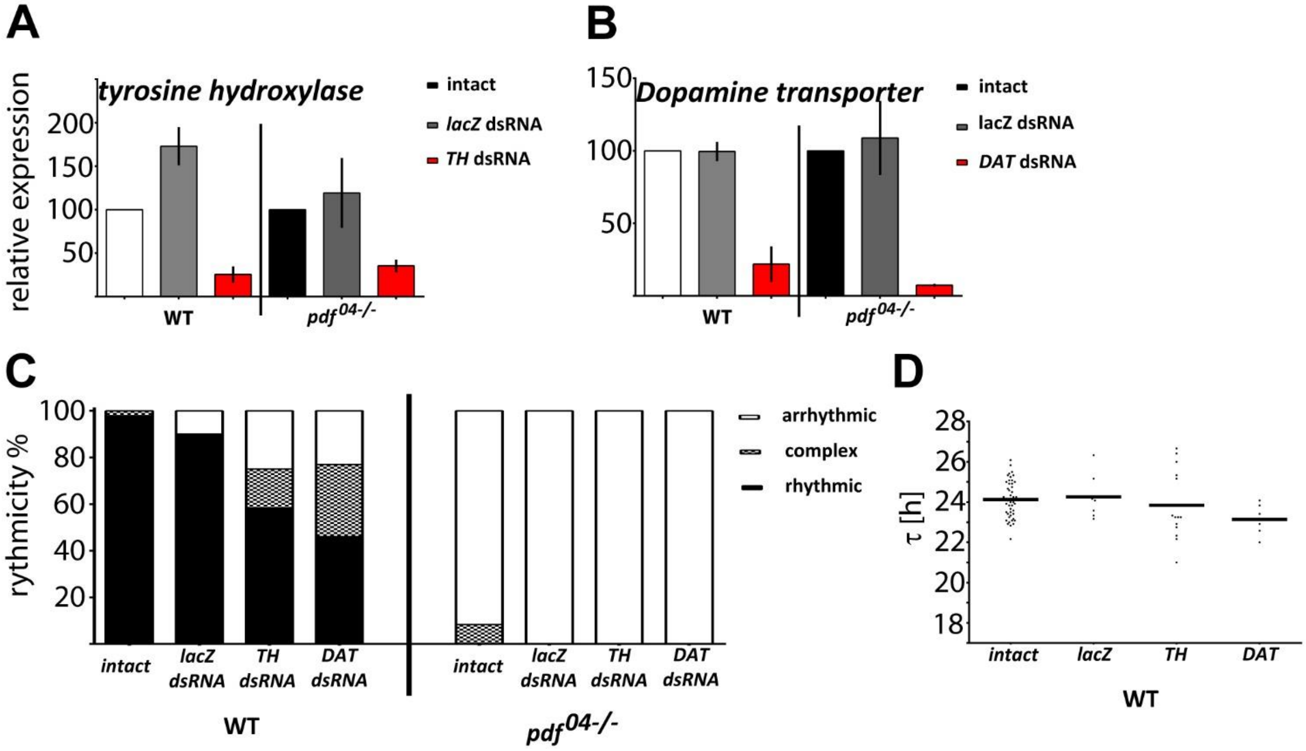

Supplementary Figure 2. (A) The efficiency of the *tyrosine hydroxylase* (*TH*) transcript downregulation by RNAi in WT and *pdf* $^{04-/-}$ mutant (Kruskal Wallis test $p<0.01$ and $p<0.05$, respectively). (B) The efficiency of the *Dopamine transporter* (*DAT*) transcript downregulation by RNAi in WT and *pdf* $^{04-/-}$ mutant (Kruskal Wallis test $p<0.05$ and $p<0.05$, respectively). (C) The rhythmicity of intact, and *lacZ*, *TH*, and *DAT* dsRNA treated WT and *pdf* $^{04-/-}$ bugs. (D) The length of the free-running of intact, and *lacZ*, *TH*, and *DAT* dsRNA treated WT bugs.
