# Supplementary material for: Pigment Dispersing Factor Is a Circadian Clock Output and Regulates Photoperiodic Response in the Linden Bug, Pyrrhocoris apterus
Source: Front Physiol. 2022 Apr 29;13:884909. doi: 10.3389/fphys.2022.884909 (PMC9099023; doi:10.3389/fphys.2022.884909)
Supplement: Supplementary file 2 [file Table1.DOCX]

Supplementary Material

**Supplementary Table 1**

Primers used to for qPCR (*q_name*) and for dsRNA templates (*ds_name*).

| gene | Forward 5’-3’ | Reverse 5’-3’ |
| --- | --- | --- |
| *q_rp49* | (Kotwica-Rolinska et al., 2017) |  |
| *q_Clk* | (Kotwica-Rolinska et al., 2017) |  |
| *q_cyc* | (Kotwica-Rolinska et al., 2017) |  |
| *q_cry-m* | (Kotwica-Rolinska et al., 2017) |  |
| *q_per* | (Kotwica-Rolinska et al., 2017) |  |
| *q_pdf* | AGATGTCTCATCACTTCCGACG | CCTTTACTAGGTCAGCAAGCCA |
| *q_TH* | ACCAATGATGCCGGTCTTAC | CGCGAAGCTTGACTACCAA |
| *q_DAT* | CAAATTATGATCACTCCTGGCTCA | GGATCAGTCTGTATCCCATTCAT |
| *ds_TH* | CAGAACTTGCTCCTTCTCATTAAG | GTACTGGGTACTTTGGAATACTCT |
| *ds_DAT* | GATATTACTTGTCCGTGGGATAAC | CAGAACCTCTCTGTTCCATAAATC |

Kotwica-Rolinska, J., Pivarciova, L., Vaneckova, H., and Dolezel, D. (2017). The role of circadian clock genes in the photoperiodic timer of the linden bug Pyrrhocoris apterus during the nymphal stage. *Physiological Entomology* 42(3)**,** 266-273. doi: https://doi.org/10.1111/phen.12197.
