# Supplementary material for: Pigment Dispersing Factor Is a Circadian Clock Output and Regulates Photoperiodic Response in the Linden Bug, Pyrrhocoris apterus
Source: Front Physiol. 2022 Apr 29;13:884909. doi: 10.3389/fphys.2022.884909 (PMC9099023; doi:10.3389/fphys.2022.884909)
Supplement: Supplementary file 3 [file DataSheet3.PDF]

Supplementary Material

>*Pyrrhocoris apterus pigment dispersing factor* (*pdf*) CDS

ATGAAAGCTATAATGGCATTTACCTTCCTCACATTCCTCTTCCTAGGATTCTTTGCAGATGTCTCATCACTTCCGACGTACA
CTTTGAATGACTTAAATGACAGGGTCATTGATAAAATTATATTGCAGACAATCAGCGAGAACGGAGGTTCACGTTGGCT
TGCTGACCTAGTAAAGGGCGAAGGACACGCACAAAAACGTAATTCGGAAATCATCAACTCACTACTCGGCATTCCAAAA
GTTGTCAATGATGCTGGCCGTTA

>*Pyrrhocoris apterus tyrosine hydroxylase* (*TH*) CDS (brain expressed)

ATGATGGCCGTAGCAGCAGCTCAGAAGAACAGGGAGATGTTCGCCATCAAGAAGTCTTACAGTATAGAGAATGGTTAT
CCTGCAAGAAGGCGTTCACTGGTCGACGACGCACGCTTCGAAACGGTCGTTGTCAAACAGACCAAGCAGAGTGTGCTT
GAAGAGGCCAGGATTAAAACCAATGATGCCGGTCTTACCGAAGAAGAGGTGATATTGTCCAATTCGGCCAACGAGAGT
CGAGAGGCCGCCGAAGCTGTCCAACGCGCCGCCTTGGTAGTCAAGCTTCGCGAGGGCATCGGATCTCTAGCAAGGATC
CTCAAGACCATCGAAGTCAACAAGGGTACAATCGTCCACCTGGAATCCAGGCCTTCCAAGGTTTCTGGAGTACAGTTCG
ATGTACTTGTTAAAATCGACATGTCCAGGCAGAACTTGCTCCTTCTCATTAAGACCCTGAGACAGAGTTCCTCTCTCGGC
GGTGTCAGTCTTCTCGGGGACAACTCCATCAACGTCAAGAACCCGTGGTTCCCGAGGCACGCGAGAGACTTGGACAAC
TGCAATCACCTTATGACAAAGTACGAACCTGAACTCGACATGAACCACCCGGGATTTGCTGACAAAGTATACAGAGAGA
GAAGGAAGCAAATCGCTAACATCGCTTTTGACTACAAATACGGTGATCCGATCCCAAGAATCAGTTACACAGCTGACGA
AGTGTCCACGTGGTCTGCAGTCTTCAACACGGTCCTTGAACTGATGCCCAAGCACTTCTGCAAGGAATACCAGGTTGTAT
TCGGTATGCTGCAGAAGGAGGGTATATTCAGAGCTGACAAGATCCCACAGCTTGAGGAGATGTCGGCCTTCCTTAAGA
AGTGTACCGGTTTCACTCTTCGTCCGGCGGCTGGACTCCTTACAGCCAGGGACTTCCTCGCAAGTCTTGCCTTCAGAGTA
TTCCAAAGTACCCAGTACGTCAGGCACACAACCACTCCGTTCCACACACCTGAACCCGACTGCATCCACGAACTCCTTGG
ACACATGCCACTATTAGCCGACCCAAGCTTCGCACAATTCTCCCAGGAAATAGGATTAGCTTCGCTTGGAGCGTCCGAC
GAGGAAATCGAAAAACTTTCAACCGTCTACTGGTTCACCGTTGAGTTCGGTCTCTGCAAGGAACATGGCAAGGTTAAGG
CATACGGAGCCGGACTTCTGTCCAGCTACGGAGAACTTCTTCACGCCATCTCCGACAAGCCCGAGCATAGGCCATTCGA
ACCATCGACAACAGCAGTACAGCCCTACCAGGATCAGGAGTATCAGCCGATTTACTTCGTCGCTGAGAGCTTCGAAGAC
GCTAAAGAGAAATTCAGGCGTTGGGTCTCTACAATGTCCAGACCGTTCGAGGTACGTTTCAACCCTCACACCCAATCGG
TGGAAGTACTTGACTCAGTGGACAGACTCGACAGCCTGGTCTCCCAGATGAACCTAGAAATGCAACACCTGACAACTGC
ACTGACCAAACTTAAGACTACCTTCGGTTAA

>*Pyrrhocoris apterus dopamine transporter* (*DAT*) CDS

ATGGAGTCTGATCGCCGGGAGACATGGTCCGGCAAGGTGGACTTCCTTCTGTCGGTCATCGGCTTCGCCGTCGACCTCG
CAAACGTATGGAGGTTCCCGTATCTCTGCTACAAGAACGGAGGCGGTGCTTTCCTTGTGCCCTACTGTATAATGCTGTTC
GTAGGAGGTATACCCCTGTTCTATATGGAGCTGGCTCTGGGACAGTTCCACAGGAAGGGAGCCATCACCTGCTGGGGC
AGGATAGTACCTCTATTTAAAGGAATAGGCTACGCTGTTGTACTAATTGCGTTTTATGTTGAATGGGCCCTACGGTTTTT
CTTTGCCTCATTCACAGATCTCCTACCGTGGACGACATGCGACAACCCATGGAACACCCCAAACTGCAGGCCGTTAGAC
GTCAGCATATGGTCTACAAATTCCAACACAACCGGAAACATTACATTCAAGAATGCATCTACCTCTGCCCCGCCTTCCGA
TAATTTCTCATCTGCTTCTTATGAATACTTTACGAGAGCAATCCTTGAACTCCATCATAGCGAGGGGCTCCACGACTTGG
GAGCTATAAAATGGGACATGGCACTTTGTCTTTTTGCTGTATACCTTATTTGCTATTTCAGTCTCTGGAAGGGTATTTCAA
CATCTGGAAAGGTGGTGTGGTTCACAGCTCTATTTCCATACGCAGTACTGCTGATATTACTTGTCCGTGGGATAACACTT
CCAGGTTCAGCCGATGGAATTAAATATTACCTCAGCCCCAATTTTTCATTAATCACTAAAGCAGAGGTGTGGGTTGACGC
CGCCACCCAAGTCTTCTTCTCGCTCGGCCCAGGTTTCGGGGTACTCCTCGCCTACGCCTCCTATAATGAATATCACAACAA
TGTCTACCAGGATGCCATTTTGACTTCGATGATAAATTCCTGTACTAGTTTCATTGCTGGTTTCGTCATATTCTCGGTTCTC
GGATACATGGCTCATGTGACCGGCAAGAACATCGCAGACGTGGCCACTGAAGGGCCGGGACTCGTGTTCATCGTCTAC
CCGGCTGCCATAGCCACGATGCCCGGTTCTATATTCTGGGCCCTCATATTCTTCATGATGCTTCTAACTCTCGGCCTCGAC
AGCTCGTTTGGTGGCTCTGAAGCAATCATTACAGCATTGAGTGATGAGTTTCCATTAATTGGAAAAAACAGGGAAATTT

TTGTAGCTTGCCTCTTTACGTTATACTTTATCGTAGGACTTGCATCATGCACTCAAGGAGGTTTTTACTTTTTTCATCTTCT
TGACCGATATGCTGCGGGATATTCCATGCTTTTTGCAGTATTTTTTGAATCAATTGCTGTTGCCTGGATTTATGGAACAGA
GAGGTTCTGTGGTGACATTAAGGACATGGTTGGCTTTAGACCTGGTTGCTACTGGAGGGTGTGCTGGAGATTTTTAGCA
CCAACATTCATTATGTTTATCATTGTTTATGGATTGCTAGCCTATGAACCATTAACTTATGGGGATTATGTATACCCAATTT
GGGCAAACGTTTTAGGCTGGGCCATTGCAATGTCGAGCATTATTATGATCCCGGCTATGGCTCTATACCAAATTATGATC
ACTCCTGGCTCATTCATGCAAAGAATGAAAATATTGACAACTCCTTGGAGAGATCACCAGACAGTTTTAGCCCGGTCTAT
GTCAATGAATGGGATACAGACTGATCCTGCCCAAATTAGGTTGACTACACCCCAAGCAACTGATCAAGTTTAA

Supplementary Figure 3 Coding sequence (CDS) of *Pyrrhocoris apterus: pigment dispersing factor* (*pdf*), brain expressed *tyrosine hydroxylase* (*TH*) and *Dopamine transporter* (*DAT*).
