# Supplementary material for: Pigment Dispersing Factor Is a Circadian Clock Output and Regulates Photoperiodic Response in the Linden Bug, Pyrrhocoris apterus
Source: Front Physiol. 2022 Apr 29;13:884909. doi: 10.3389/fphys.2022.884909 (PMC9099023; doi:10.3389/fphys.2022.884909)
Supplement: Supplementary file 4 [file Table2.DOCX]

Supplementary Material

**Supplementary Table 2**

Summary of the gene knockdown effect on the behavioral rhythmicity

| **Effect of *pdf* mutation on the behavioral rhythmicity in LD and DD** | | | | | | | | |
| --- | --- | --- | --- | --- | --- | --- | --- | --- |
| genotype | LD | N | LD  [%]  rhythmic | DD  [%]  rhythmic | DD  [%]  complex | DD  [%]  arrhythmic | tau [h] | SEM  +/- |
| WT (*pdf*) | 18:6 | 44 | 97.7 | 95.5 | 0 | 2.5 | 23.80 | 0.15 |
| *pdf ^RK +/+^* | 18:6 | 15 | 93.3 | 100 | 0 | 0 | 23.60 | 0.13 |
| *pdf ^RK+/-^* | 18:6 | 15 | 100 | 100 | 0 | 0 | 23.76 | 0.14 |
| *pdf ^RK -/-^* | 18:6 | 22 | 95.5 | 100 | 0 | 0 | 24.45 | 0.16 |
| *pdf^05+/+^* | 18:6 | 17 | 94.1 | 88.2 | 0 | 11.8 | 23.25 | 0.21 |
| *pdf^05+/-^* | 18:6 | 23 | 91.3 | 86.9 | 0 | 13.1 | 23.90 | 0.19 |
| *pdf^05-/-^* | 18:6 | 50 | 80 | 2 | 6 | 92 | 17.42 | - |
| *pdf^04+/+^* | 18:6 | 32 | 100 | 62.5 | 9.4 | 28.1 | 24.26 | 0.17 |
| *pdf^04+/-^* | 18:6 | 68 | 100 | 50 | 7.4 | 42.6 | 25.66 | 0.30 |
| *pdf^04-/-^* | 18:6 | 39 | 68.6 | 2.6 | 5.1 | 92.3 | 19.83 | - |
| *pdf^07+/+^* | 18:6 | 19 | 100 | 100 | 0 | 0 | 23.34 | 0.19 |
| *pdf^07+/-^* | 18:6 | 54 | 94.4 | 98.1 | 1.9 | 0 | 23.60 | 0.38 |
| *pdf^07-/-^* | 18:6 | 79 | 79.2 | 20.8 | 8.3 | 70.9 | 20.38 | 0.48 |
| *pdf ^03L+/+^* | 18:6 | 32 | 87.5 | 81.2 | 3.1 | 15.7 | 23.96 | 0.25 |
| *pdf ^03L+/-^* | 18:6 | 112 | 83.9 | 85.6 | 0 | 14.4 | 23.81 | 0.14 |
| *pdf ^03L-/-^* | 18:6 | 95 | 84.2 | 4.2 | 3.2 | 92.6 | 21.83 | 1.68 |
| **Effect of *cry* mutation on the behavioral rhythmicity in LD and DD** | | | | | | | | |
| genotype | LD | N | LD  [%]  rhythmic | DD  [%]  rhythmic | DD  [%]  complex | DD  [%]  arrhythmic | tau [h] | SEM  +/- |
| WT (*cry-m*) | 18:6 | 24 | 100 | 54.2 | 4.2 | 41.6 | 24.70 | 0.43 |
| *cry-m^04-/-^* | 18:6 | 29 | 96.6 | 3.4 | 17.2 | 79.4 | 22.25 | - |
| *cry-m^9in-/-^* | 18:6 | 29 | 86.2 | 10.3 | 13.8 | 75.9 | 19.17 | 0.87 |
| **Effect of dopamine signaling disruption on the behavioral rhythmicity of WT and *pdf^04-/-^* mutants in DD** | | | | | | | | |
| Genotype  (treatment) | LD | N | LD  [%]  rhythmic | DD  [%]  rhythmic | DD  [%]  complex | DD  [%]  arrhythmic | tau [h] | SEM  +/- |
| WT  (intact) | 18:6 | 48 | not analyzed | 97.9 | 2.1 | 0 | 24.1 | 0.13 |
| WT  (*lacZ* dsRNA) | 18:6 | 10 | not analyzed | 90.0 | 0 | 10.0 | 24.2 | 0.33 |
| WT  (*TH* dsRNA) | 18:6 | 24 | not analyzed | 58.3 | 16.7 | 25.0 | 23.9 | 0.46 |
| WT  (*DAT* dsRNA) | 18:6 | 13 | not analyzed | 46.2 | 30.7 | 23.1 | 23.1 | 0.32 |
| *pdf^04-/-^*  (intact) | 18:6 | 12 | not analyzed | 0 | 8.3 | 91.7 | - | - |
| *pdf^04-/-^*  (*lacZ* dsRNA) | 18:6 | 11 | not analyzed | 0 | 0 | 100 | - | - |
| *pdf^04-/-^*  (*TH* dsRNA) | 18:6 | 22 | not analyzed | 0 | 0 | 100 | - | - |
| *pdf^04-/-^*  (*DAT* dsRNA) | 18:6 | 9 | not analyzed | 0 | 0 | 100 | - | - |
| **Behavioral activity of WT and *pdf^04-/-^* in different photo-regimes** | | | | | | | | |
| genotype | LD | N | LD  [%]  rhythmic | DD |  |  |  |  |
| WT | 18:6 | 43 | 100 | not analyzed |  |  |  |  |
| WT | 15:9 | 28 | 96.4 | not analyzed |  |  |  |  |
| WT | 12:12 | 47 | 89.3 | not analyzed |  |  |  |  |
| WT | 9:15 | 25 | 92 | not analyzed |  |  |  |  |
| *pdf^04-/-^* | 18:6 | 27 | 76.7 | not analyzed |  |  |  |  |
| *pdf^04-/-^* | 15:9 | 21 | 85.7 | not analyzed |  |  |  |  |
| *pdf^04-/-^* | 12:12 | 43 | 79.5 | not analyzed |  |  |  |  |
| *pdf^04-/-^* | 9:15 | 27 | 81.5 | not analyzed |  |  |  |  |
| **Behavioral activity of WT and *pdf^04-/-^* during 5 days in LD18:6 and following 10 days in constant conditions** | | | | | | | | |
| Genotype  (photo-regime) | LD | N | LD  [%]  rhythmic | Constant  conditions  [%]  rhythmic | Constant  conditions [%]  complex | Constant  conditions [%]  arrhythmic | tau [h] | SEM  +/- |
| WT  (LD-DD) | 18:6 | 14 | 96.4 | 100 | 0 | 0 | 24.33 | 0.24 |
| WT  (LD-LL) | 18:6 | 18 | 94.4 | 60 | 0 | 40 | 22.76 | 0.55 |
| *pdf^04-/-^*  *(*LD-DD) | 18:6 | 39 | 68.6 | 2.6 | 5.1 | 92.3 | 19.83 | - |
| *pdf^04-/-^*  (LD-LL) | 18:6 | 45 | 77.8 | 6.7 | 0 | 93.3 | 19.21 | 1.85 |
