# Supplementary material for: Pigment Dispersing Factor Is a Circadian Clock Output and Regulates Photoperiodic Response in the Linden Bug, Pyrrhocoris apterus
Source: Front Physiol. 2022 Apr 29;13:884909. doi: 10.3389/fphys.2022.884909 (PMC9099023; doi:10.3389/fphys.2022.884909)
Supplement: Supplementary file 5 [file DataSheet1.pdf]

Supplementary Material

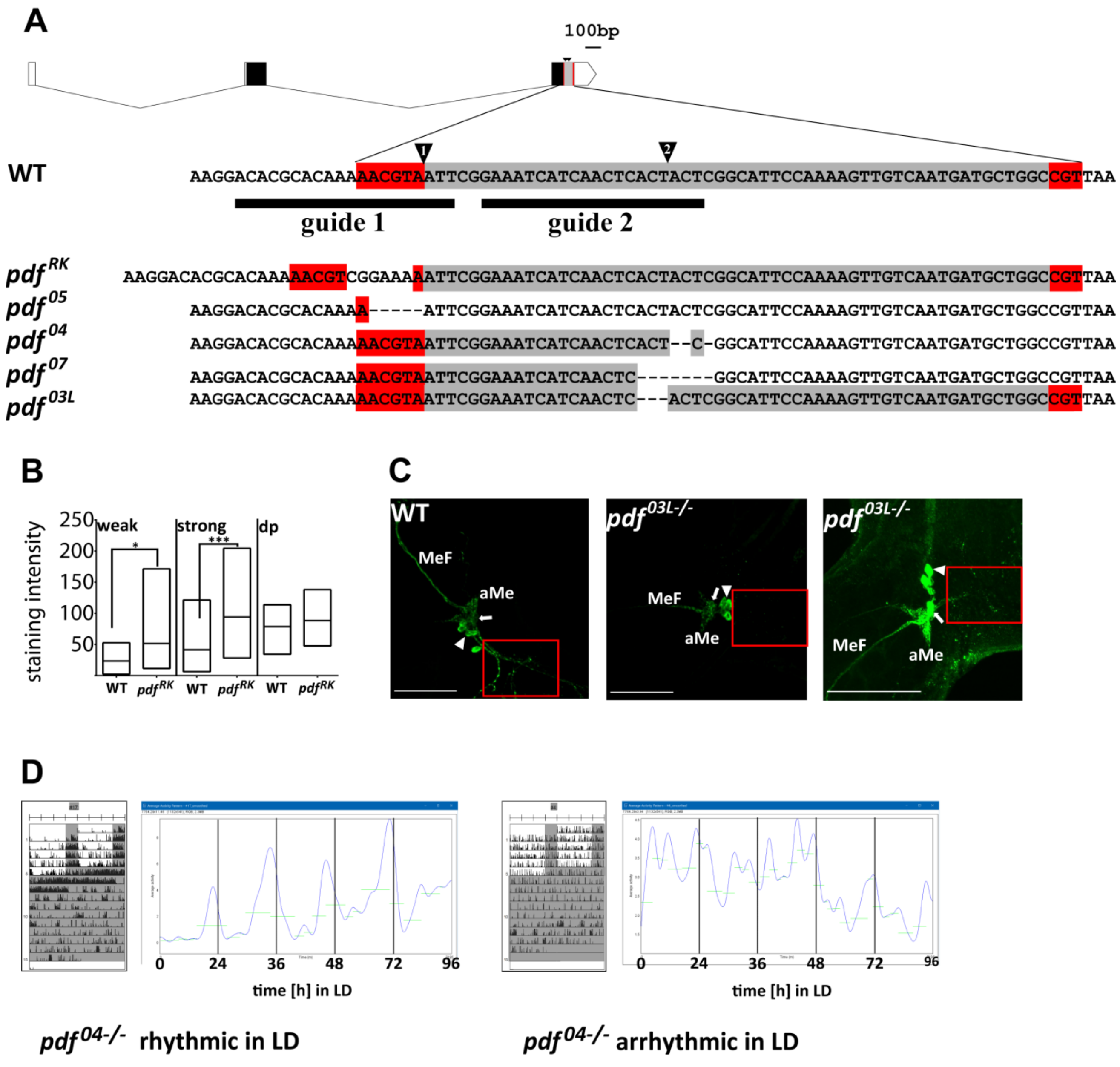

Supplementary Figure 1. (A) Schematic representation *P. apterus pdf* gene structure (online tool Exon-Intron Graphic Maker http://wormweb.org/exonintron) and detailed nucleotide sequence encoding PDF active peptide (grey rectangle) targeted by CRISPR/Cas9. The red rectangles flanking PDF nucleotide sequence encode KR and R predicted to be prohormone convertase cleavage sites in insects. Position of guides is marked by the black horizontal line and predicted Cas9 cut sites are depicted as black numbered triangles. 5 unique lines were obtained by CRISPR/Cas9 mutagenesis

(description in the manuscript). (B) PDF-immunostaining intensity in weakly and strongly stained cells and dorsal protocerebrum arborizations is higher in *pdf* $^{RK-/-}$ mutants than in WT (Mann Whitney test, weakly stained cells (*) $p<0.05$ strongly stained cells (***) $p<0.001$, dorsal protocerebrum $p>0.05$). (C) Representative image of PDF staining in the optic lobe of WT (on the left) and *pdf* $^{03L-/-}$ mutant (in the middle) taken with the same confocal settings. The staining intensity of PDF in accessory medulla (aMe) ramifications, and in medulla Fiber (MeF) is weaker in *pdf* $^{03L-/-}$. Weakly (arrow) and strongly (arrowhead) PDF stained cells are present in *pdf* $^{03L-/-}$. The image of the optic lobe of the *pdf* $^{03L-/-}$ mutant with increased laser strength (on the right) shows that *pdf* $^{03L-/-}$ lacks the PDF-ir neuronal processes leading towards dorsal protocerebrum (red rectangle). Scale bar = 100μm. (D) Representative actograms of *pdf* $^{04-/-}$ scored as "rhythmic in LD" (on the left) and "arrhythmic in LD" (on the right). Smoothed activity profile of the bugs during 5 days in LD cycles is presented next to the corresponding actograms. The "rhythmic in LD" activity profile shows one clear peak of activity on every day. The "arrhythmic in LD" activity profile shows several peaks of activity during 24h. Grey areas on actograms depict darkness.
