# Supplementary material for: Pigment Dispersing Factor Is a Circadian Clock Output and Regulates Photoperiodic Response in the Linden Bug, Pyrrhocoris apterus
Source: Front Physiol. 2022 Apr 29;13:884909. doi: 10.3389/fphys.2022.884909 (PMC9099023; doi:10.3389/fphys.2022.884909)
Supplement: Supplementary file 6 [file Table3.docx]

Supplementary Material

**Supplementary Table 3**

Reproductive status of *pdf* and *cry-m* mutants in short day conditions (SD)

| genotype | n | % diapausing |
| --- | --- | --- |
| WT | 35 | 100 |
| *pdf^/ RK +/+^* | 38 | 89.5 |
| *pdf ^RK+/-^* | 113 | 96.5 |
| *pdf ^RK -/-^* | 59 | 98.3 |
| *pdf ^05+/+^* | 39 | 100 |
| *pdf ^05+/-^* | 103 | 98.1 |
| *pdf ^05-/-^* | 29 | 65.5 |
| *pdf ^04+/+^* | 18 | 100 |
| *pdf ^04+/-^* | 53 | 100 |
| *pdf ^04-/-^* | 34 | 29.4 |
| *pdf ^07+/+^* | 23 | 91.3 |
| *pdf ^07+/-^* | 43 | 90.7 |
| *pdf ^07-/-^* | 23 | 13.0 |
| *pdf ^03L+/+^* | 36 | 88.9 |
| *pdf ^03L+/-^* | 93 | 94.6 |
| *pdf ^03L-/-^* | 25 | 20 |
|  |  |  |
| *cry-m^04+/+^* | 77 | 100 |
| *cry-m^04+/-^* | 147 | 100 |
| *cry-m^04-/-^* | 67 | 7.5 |
| *cry-m^9in+/+^* | 89 | 100 |
| *cry-m^9in+/-^* | 157 | 100 |
| *cry-m^9in-/-^* | 52 | 6.0 |
